# Neuropathological assessment of the olfactory bulb and tract in individuals with COVID-19

**DOI:** 10.1186/s40478-024-01761-8

**Published:** 2024-05-03

**Authors:** Nathalie A. Lengacher, Julianna J. Tomlinson, Ann-Kristin Jochum, Jonas Franz, Omar Hasan Ali, Lukas Flatz, Wolfram Jochum, Josef Penninger, aSCENT-PD Investigators, Benjamin Arenkiel, Zhandong Liu, Brit Mollenhauer, Josef Penninger, Maxime Rousseaux, Armen Saghatelyan, Natalina Salmaso, Christine Stadelmann, Michael G. Schlossmacher, Julianna J. Tomlinson, John M. Woulfe, Christine Stadelmann, John M. Woulfe, Michael G. Schlossmacher

**Affiliations:** 1 Neuroscience Program, Ottawa Hospital Research Institute, Ottawa, ON Canada; 2 Institute of Pathology, Kantonsspital St. Gallen, St. Gallen, Switzerland; 3 Institute of Immunobiology, Kantonsspital St. Gallen, St. Gallen, Switzerland; 4 Neuropathology Institute, University of Goettingen Medical Centre, Goettingen, Germany; 5 Department of Life Sciences, University of British Columbia, Vancouver, BC Canada; 6 Department of Pathology and Laboratory Medicine, The Ottawa Hospital, Ottawa, ON Canada; 7 Department of Dermatology, University Hospital Tübingen, Tübingen, Germany; 8 Division of Neurology, Department of Medicine, The Ottawa Hospital, Ottawa, ON Canada; 9 Aligning Science Across Parkinson’s (ASAP) Collaborative Research Network, Chevy Chase, MD 20815 USA

**Keywords:** COVID-19, Olfaction, Hyposmia, Neurodegeneration, Microglia, Histiocytes, Inflammation, Amyloidosis, Tauopathy, Synucleinopathy, Anterior olfactory nucleus

## Abstract

The majority of patients with Parkinson disease (PD) experience a loss in their sense of smell and accumulate insoluble α-synuclein aggregates in their olfactory bulbs (OB). Subjects affected by a SARS-CoV-2-linked illness (COVID-19) also frequently experience hyposmia. We previously postulated that microglial activation as well as α-synuclein and tau misprocessing can occur during host responses following microbial encounters. Using semiquantitative measurements of immunohistochemical signals, we examined OB and olfactory tract specimens collected serially at autopsies between 2020 and 2023. Deceased subjects comprised 50 adults, which included COVID19 + patients (n = 22), individuals with Lewy body disease (e.g., PD; dementia with Lewy bodies (n = 6)), Alzheimer disease (AD; n = 3), and other neurodegenerative disorders (e.g., progressive supranuclear palsy (n = 2); multisystem atrophy (n = 1)). Further, we included neurologically healthy controls (n = 9), and added subjects with an inflammation-rich brain disorder as neurological controls (NCO; n = 7). When probing for microglial and histiocytic reactivity in the anterior olfactory nuclei (AON) by anti-CD68 immunostaining, scores were consistently elevated in NCO and AD cases. In contrast, microglial signals on average were not significantly altered in COVID19 + patients relative to healthy controls, although anti-CD68 reactivity in their OB and tracts declined with progression in age. Mild-to-moderate increases in phospho-α-synuclein and phospho-tau signals were detected in the AON of tauopathy- and synucleinopathy-afflicted brains, respectively, consistent with mixed pathology, as described by others. Lastly, when both sides were available for comparison in our case series, we saw no asymmetry in the degree of pathology of the left versus right OB and tracts. We concluded from our autopsy series that after a fatal course of COVID-19, microscopic changes in the rostral, intracranial portion of the olfactory circuitry -when present- reflected neurodegenerative processes seen elsewhere in the brain. In general, microglial reactivity correlated best with the degree of Alzheimer’s-linked tauopathy and declined with progression of age in COVID19 + patients.

**Supplementary Information:**

The online version contains supplementary material available at 10.1186/s40478-024-01761-8.

## Introduction

Parkinson disease (PD) is traditionally characterized clinically by extrapyramidal motor dysfunction and pathologically by the progressive degeneration of dopamine producing neurons in the Substantia nigra. Over the past two decades, this nigral- and moto-centric view of PD has undergone a revision with the recognition that the disorder is also characterized by a wide variety of non-motor symptoms referable not only to brain pathology but to the peripheral nervous system as well [1]. Indeed, these symptoms may predate the onset of extrapyramidal motor dysfunction by decades [1]. Accordingly, a histopathological hallmark of typical, late-onset PD, namely intracellular α-synuclein (αSyn) aggregation in the form of Lewy bodies and Lewy neurites, has been described in extra-nigral sites within the brain as well as within peripheral organs, often prior to their emergence in the Substantia nigra. Braak and Del Tredici [2] provided pathological evidence for a dynamic spatiotemporal sequence of αSyn pathology in the brain. According to their hypothesis, the OBs and dorsal motor nuclei of the vagus nerve represent the earliest CNS sites in which αSyn aggregation occurs. Implicit in the Del Tredici-Braak model is the phenomenon of ‘spreading’ pathology, whereby -once initiated at those sites- the aggregation process is transmitted trans-synaptically to other nuclei including the Substantia nigra, resulting in the well-recognized motor features of the disease [3]. Consistent with this hypothesis, hyposmia is one of the earliest and most frequent non-motor signs of PD [1, 4], which accords with the presence of Lewy pathology in the OBs as well as in higher order olfactory structures [2]. As a potential trigger of αSyn aggregation in the olfactory system and enteric nervous system, Braak and Del Tredici invoked an infectious, possibly viral, pathogen [3]. In this context, their hypothesis is compatible with the growing body of evidence implicating microbial encounters as risk factors for neurodegenerative disorders in later years (reviewed in [5]).

Subsequent to its discovery in Wuhan, China in late 2019, coronavirus-linked disease 2019 (COVID-19), caused by severe acute respiratory syndrome coronavirus-2 (SARS-CoV-2), spread rapidly throughout the world, culminating in a global pandemic that imposed substantial human suffering and loss of life as well as unprecedented social and economic consequences [6]. Olfactory dysfunction in the form of anosmia, hyposmia and parosmia were described as a common symptom during the pandemic, affecting 30–70% of all patients with confirmed COVID-19 [7]. Abnormal olfaction usually occured early in the course of infection, presenting as the first symptom in approximately 12% of all patients [8]. Although most patients recovered their sense of smell gradually, often within 3–4 weeks [9, 10], some suffered from persistent impairment, suggesting severe and/or permanent damage to components within the olfactory circuitry [9, 11]. Several possible processes have been invoked as mechanisms underlying olfactory dysfunction in COVID-19, including direct infection of olfactory sensory neurons (OSNs) in the nasal cavity [12] as well as primary infection of sustentacular cells with secondary injury to OSNs. In accordance with the latter, several investigators have noted the absence of the SARS-CoV-2 target receptor, angiotensin converting enzyme-2, on neuronal cells, and instead proposed infection and damage to non-neuronal support cells in the olfactory epithelium [13, 14]. Regardless of the cellular target, there is histopathological evidence that infection by SARS-CoV-2 can confer effects onto the CNS in the form of local inflammation, axonal pathology and microvascular changes in the OB and olfactory tracts (OT), including OB deafferentation [11, 15].

Moreover, a recent population-based neuroimaging study in survivors of a SARS-CoV-2 infection revealed chronically altered volumes of olfaction circuitry-associated areas in the brain. It provided evidence that the potential impact of SARS-CoV-2 on scent processing extends beyond the OT to impose lasting neurodegenerative and/or remodeling changes in the central olfactory circuitry [16].

That COVID-19 and PD share olfactory dysfunction as an early clinical sign is intriguing in the context of the Braak-Del Tredici hypothesis, which implicates exposure to one or several pathogens in disease initiation. Moreover, the presence of abundant αSyn in the mammalian olfactory epithelium and mucosa [17] renders the nasal passages a plausible site for a disease-initiating interaction between airborne pathogens, such as RNA viruses, and αSyn, a highly expressed brain protein that is prone to misfolding. Consistent with this possibility, animal studies have revealed an upregulation and accumulation of αSyn in response to intranasal SARS-CoV-2 administration [18, 19]. It is believed that the impact of the virus on αSyn metabolism may be imposed either via a direct interaction intracellularly or may be related to innate, immunomodulatory functions of extracellular αSyn [20]. With respect to the former, heparin-binding sites on the SARS-CoV-2 spike protein may act as facilitator to seed the aggregation of other heparin-binding proteins, including αSyn, as well as amyloid β-peptide and tau [21]. Further, SARS-CoV-2 proteins are capable of forming amyloid aggregates themselves [22], possibly providing a nidus for the aggregation of neurodegeneration-associated proteins. Alternatively, or in addition, αSyn has been demonstrated to function as an innate, anti-viral factor [17, 23]. In theory, its upregulation in response to SARS-CoV-2 infection could predispose it to aggregation, thereby putting COVID-19 patients at higher risk of developing PD-relevant changes [24]. Of note, few cases of parkinsonism associated with SARS-CoV-2 infection in humans have been documented since the beginning of the pandemic [25–27]. In contrast, a substantial number of patients with established PD experienced worsening of their symptoms during and after recovery from COVID-19 illness [28].

These lines of evidence implicate SARS-CoV-2 infection as a possible trigger for the initiation of PD-linked pathology. However, the scarcity of histopathological studies interrogating inflammatory and neurodegenerative changes and specifically probing for αSyn aggregation in the olfactory pathway following SARS-CoV-2 infection, represents a gap in our understanding of the relationship between the two disorders. In the present autopsy study, we employ an immunohistochemical approach to determine the nature and extent of inflammation-related changes and of neurodegeneration within rostral olfactory structures, specifically the OB and OT, among a cohort of individuals that died of COVID-19-related complications versus those with neurodegenerative synucleinopathies, including PD, dementia with Lewy bodies (DLB), and multisystem atrophy (MSA). In addition, in light of reports that COVID-19 may predispose to Alzheimer disease (AD) [29], we have included participants with dementia as well as another tauopathy, progressive supranuclear palsy (PSP). We compared microglial/histiocytic and neurodegenerative changes in the OB and OT of these groups with a series of COVID-19-negative subjects without a neurodegenerative disease. Among the latter, we included as a further control a subset of participants with primary inflammatory disorders of the brain.

## Methods

### Study subjects

Study subjects were recruited at two sites: The Ottawa Hospital in Ottawa, Ontario, Canada and the Kantonsspital St. Gallen, in St. Gallen, Switzerland. Ethics approval was obtained from the Ottawa Health Science Network Research Ethics Board (#20120963-01H) and the review board for the Kantonsspital St. Gallen (Ethikkommission Ostschweiz, Projekt-ID 2021-00678).

Characteristics of the study groups are summarized in Table 1. We examined tissue from 50 individuals: 16 COVID-19-negative controls, including 9 neurologically healthy participants (HCO) and 7 with an inflammatory CNS disorder (referred to as neurological controls, NCO), 22 subjects who died from SARS-CoV-2 infection-related complications (referred to as COVID19 +), 6 participants with Lewy Body disease (LBD), three with AD, and three with other neurodegenerative diseases (OND; including PSP, n = 2; MSA, n = 1). The age of subjects ranged from 51 to 90 years. All COVID19 + patients were clinically free of a previously identified neurodegenerative illness based on medical chart review during their admission to the hospital, with the exception of one person, who demonstrated signs of parkinsonism, but without a formal diagnosis by a neurologist. Of note, olfactory function was not routinely measured in study participants; as documented in the chart, one COVID19 + patient reported the loss of sense of smell on admission. Details with respect to clinical diagnoses, respiratory complications (if any), causes of death and the subjects’ pathological findings can be found in Additional file 1: Table S1.

**Table 1:** Demographics of study group

	N	Age (mean ± SD)	Age (yrs, range)	Sex		PMI (hrs, mean ± SD)
				M	F	
Controls	16	71.8 ± 8.6	55–84	7	9	68.3 ± 87.0
Healthy control	9	72.8 ± 7.5	59–80	4	5	52.1 ± 24.3
Neurological control	7	70.6 ± 10.5	55–84	3	4	89.1 ± 131.2
COVID19 + ​	22	74.8 ± 12.1	51–89	13	9	43.9 ± 25.2
Lewy body disease	6	78.7 ± 6.7	72–90	6	0	104.0 ± 111.2
Alzheimer disease	3	69.7 ± 15.3	52–78	1	2	56.0 ± 55.4
Other neurodegenerative disease	3	78.0 ± 3.6	74–79	3	0	112.0 ± 113.4

### Tissue processing

Tissues were removed and processed in the Departments of Pathology at The Ottawa Hospital and the Kantonsspital St. Gallen according to routine procedures for *post mortem* brain collection. Following their removal at autopsy, brain specimens were fixed typically for 10–14 days, rarely as long as 53 days, in 20% neutral buffered formalin; the duration of fixation for each case can be found in Additional file 1: Table S1. Following fixation, OBs and OT were dissected from the brain (unless they had already been separated at the time of brain removal) and embedded in paraffin.

### Immunohistochemistry

Sections were cut at 5 µm and mounted onto coated slides. Slides were deparaffinized in Citrisolv and rehydrated through a series of decreasing ethanol concentrations. Endogenous peroxidase activity was quenched with 0.3% hydrogen peroxide in methanol, followed by heat-induced antigen retrieval with citrate buffer, pH 6.0. For amyloid-β peptide staining, slides were also incubated in 98% formic acid for 5 min at room temperature. To reduce non-specific binding, sections were blocked in 10% goat serum in PBS-T (PBS + 0.1% Triton-X-100 + 0.05% Tween-20) for 30 min at room temperature. Sections were incubated overnight at 4 °C in primary antibodies diluted in 5% goat serum in PBS-T. Primary antibodies included those to: p-αSyn, pSyn#64 (Wako, cat# 015-25191, 1:500); LB509 (Biolegend, cat# 807702, 1:10,000); amyloid-β peptide, 6E10 (Biolegend cat# 803001, 1:1000); KiM-1p (CD68; prepared in the Stadelmann lab; Radzun et al. [30]; 1:50); and SARS Nucleocapsid protein (Novus, cat# NB100-56576, 1:250). Biotinylated, secondary antibody anti-mouse or anti-rabbit IgG (H + L), made in goat (Vector Labs, BA-9200 or BA-1000) was diluted to 1:225, and sections were incubated for 1 h at room temperature. The signal was amplified with VECTASTAIN® Elite® ABC HRP Kit (Vector Labs, PK-6100) for 1 h at room temperature and visualized using 3′3-diaminobenzidine (Sigma, SIGMAFAST™ DAB, D4293). Samples were counterstained with Harris’ Modified Hematoxylin stain and dehydrated through a series of increasing ethanol concentration solutions and Citrisolv. Permount Mounting Medium (Fisher Scientific, SP15-100) was used for mounting, and developed slides were dried and scanned for visualization. The detailed immunohistochemistry protocol can be found at: dx.doi.org/10.17504/protocols.io.kqdg3p7mql25/v1.

Immunohistochemical staining for p-tau was performed in the Louise Pelletier Histopathology Core Facility in The Department of Pathology and Laboratory Medicine at The University of Ottawa (RRID: SCR_021737) using the Bond Polymer Refine Detection Kit (DS9800) with the Leica Bond™ system. Sections were deparaffinized and incubated using a 1:2,500 dilution of mouse p-tau antibody (#MN1020, AT8 clone; ThermoFisher) for 15 min at room temperature and detected using an HRP conjugated compact polymer system. Slides were then stained using DAB as the chromogen, counterstained with Hematoxylin, mounted and cover slipped.

All slides were digitized using a Zeiss Axio Scan.Z1 Slide Scanner at 20X resolution. Visualization of the digitized images across sites was performed using an *in-house* Omero Server software 5.6.6 (OMERO, RRID:SCR_002629, http://www.openmicroscopy.org/site/products/omero) [31] and immunohistochemistry figures were created using an *in-house* Omero Figure software v6.0.0 (https://www.openmicroscopy.org/omero/figure/). Additional details of reagents can be found in Additional file 1: Table S2.

### Quantification

**Fig. 1:**
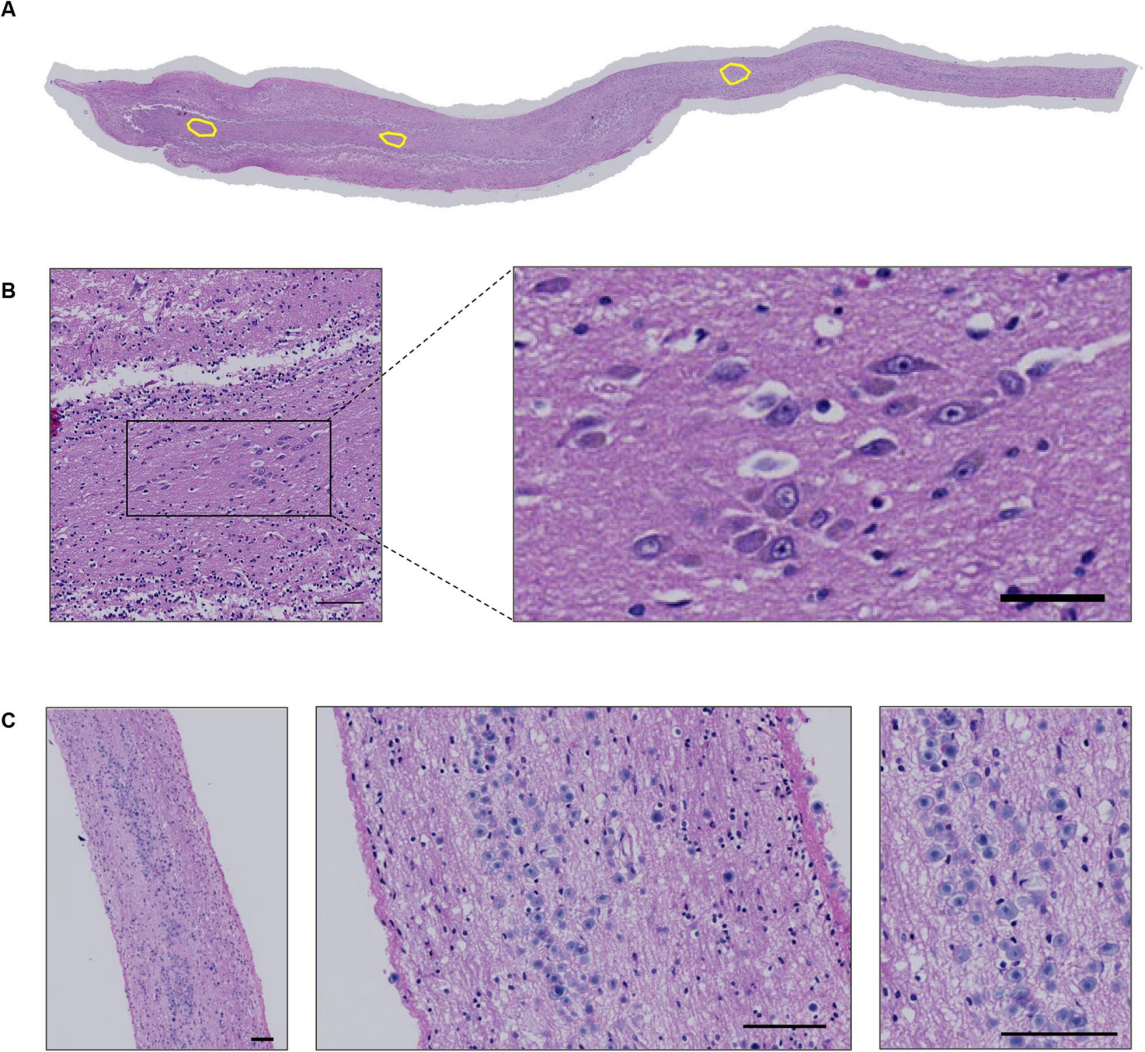
Overview of anatomical structures in the human olfactory bulb and tract. **A** Hematoxylin and eosin (H&E)-stained section of a human olfactory bulb and tract [case #23] revealing three intrafascicular anterior olfactory nuclei (AON), as outlined in yellow; in **B** at higher magnification the most rostrally located AON is shown. **C** H&E-stained sections showing corpora amylacea (in blue) located in the subpial area of the human olfactory tract. Scale bars represent 100 µM

One section from each OB/OT was stained and analysed for each marker. In preliminary studies, it was evident that staining for neurodegenerative proteins was confined predominantly to the anterior olfactory nuclei (AON); thus, semi-quantitative scoring of immunoreactivity and intensity of inflammatory as well as neurodegenerative changes were focused on these nuclei, visually defined as groups of larger neurons lying on a neuropilic background (Fig. 1). One section for each case was stained for CD68 reactivity (Kim-1P) as a surrogate marker for inflammatory changes [32]. For CD68 and neurodegeneration-focused immunostaining, semi-quantitative analyses were performed using a scale from 0 to 5, corresponding to increasing densities of DAB-positive reactivity. Pathological scores were confirmed by a second reader. For AD-associated pathology, the AxBxCx scoring system was applied to hippocampus sections as per Hyman et al. in *Alzheimer’s Dementia* 2012;8:1–13. Additional file 1: Tables S3, S4, S5, S6 and S7 indicate the number of AONs analyzed per section, which tissue region was available for each case, as well as the score assigned for each protein marker used. The dataset is also available at doi.org/10.5281/zenodo.10776590.

### Statistical analyses

Statistical analyses were performed using GraphPad Prism version 10 (GraphPad Prism, RRID: SCR_002798, www.graphpad.com). Differences between groups were determined using the Kruskall-Wallis test followed by Dunn’s post-hoc analysis. Correlation between staining score and age was determined using Pearson’s correlation tool. For all statistical analyses, a cut-off for significance was set at 0.05. Data are displayed with *p* values represented as *p < 0.05, **p < 0.01, ***p < 0.001, and ****p < 0.0001.

## Results

Hematoxylin and eosin (HE)-stained sections of the OB and attached OT are shown in Fig. 1A and B**.** These are representative of all tissue sections used in the analyses, and they indicate the location and appearance of the AON. As expected, in cases from older individuals, abundant corpora amylacea were often observed along white matter structures within the OT and were concentrated in the subpial neuropil (Fig. 1C).

When we tested for SARS-CoV2 reactivity in sections of COVID19 + patients in our autopsy series using a specific antibody against its nucleocapsid protein, we did not detect any signal for the viral protein in any of the OB and OT sections analyzed under these conditions. In contrast, the same antibody readily detected the nucleocapsid protein in sustentacular cells of the olfactory epithelium in mice nasally inoculated with a mouse-adapted variant of SARS-CoV-2 (not shown) [33].

**Fig. 2:**
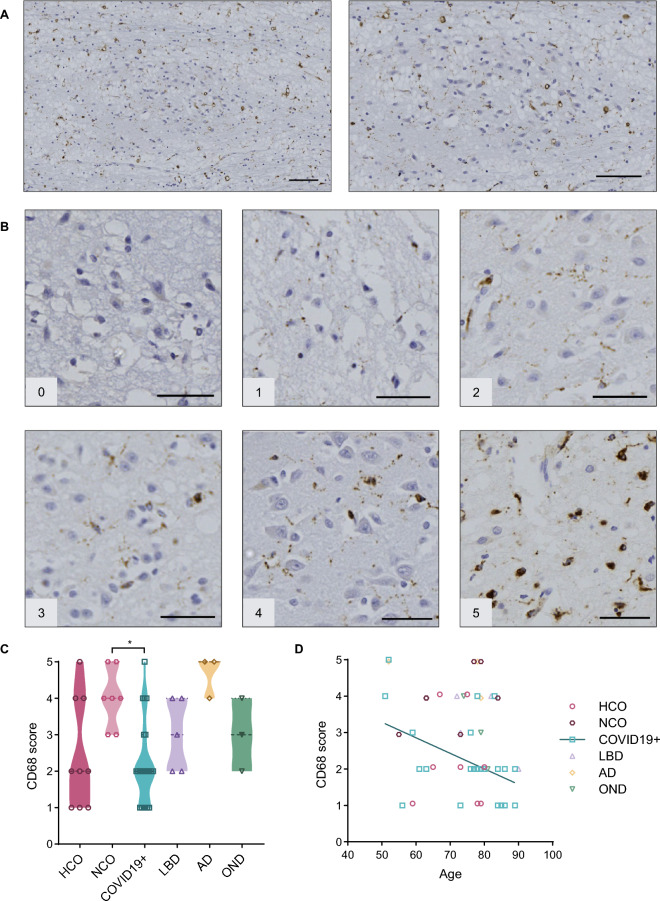
Anti-CD68 reactivity in the anterior olfactory nucleus. **A** Example of immunohistochemical staining for CD68 in the human olfactory bulb [case #10]. Scale bars represent 100 µM. **B** Representative images of semi-quantitative scoring of staining, with scale from 0 to 5, in the AON. Scale bars represent 50 µM. **C **Average staining score for each diagnostic group; filled triangle in the OND group indicates a multisystem atrophy case. **D** Scatter plot showing correlation between age and CD68 score for all groups. Significance was determined using Kruskal–Wallis test with Dunn's post-hoc, where * indicates p ≤ 0.05 (**C**) and Pearson's correlation where r = − 0.4494 (**D**). Straight line in D denotes correlation for COVID19 + cases. HCO denotes neurologically healthy controls; NCO, neurological controls; LBD, Lewy body diseases; AD, Alzheimer disease; OND, other neurodegenerative diseases

The degree of tissue inflammation-associated cellular responses was assessed using anti-CD68 (Kim-1P) immunoreactivity for the detection of histiocytes and microglia [32]. Positive CD68-immunoreactive cells were observed throughout the OB and OT (Fig. 2A). To correlate these signals with neurodegenerative changes, we focused our semi-quantitative analysis of CD68 reactivity on the AON. As indicated in Fig. 2C, all autopsy cases exhibited some degree of positive staining (a score of 1, or higher), regardless of disease status. For the purpose of comparing these microglia-related changes, we initially divided the non-neurodegenerative controls into healthy controls (HCO) and those whose diagnosis at autopsy involved an inflammatory condition of the CNS (neurological controls; NCO). Because statistically there was no significant difference in CD68 reactivity scores between HCO and NCO brains, these brains were grouped together as controls in subsequent analyses. Sections of AON from AD brains generated the highest average CD68 reactivity score (4.7) in the OB and OT, followed by the NCO group (average score, 4). COVID19 + cases had significantly lower CD68 staining scores than the NCO group (Fig. 2C). Interestingly, microglial and histiocytic activation scores based on CD68 staining correlated negatively (R = − 0.4494) with progression of age in COVID19 + patients (Fig. 2D).

**Fig. 3:**
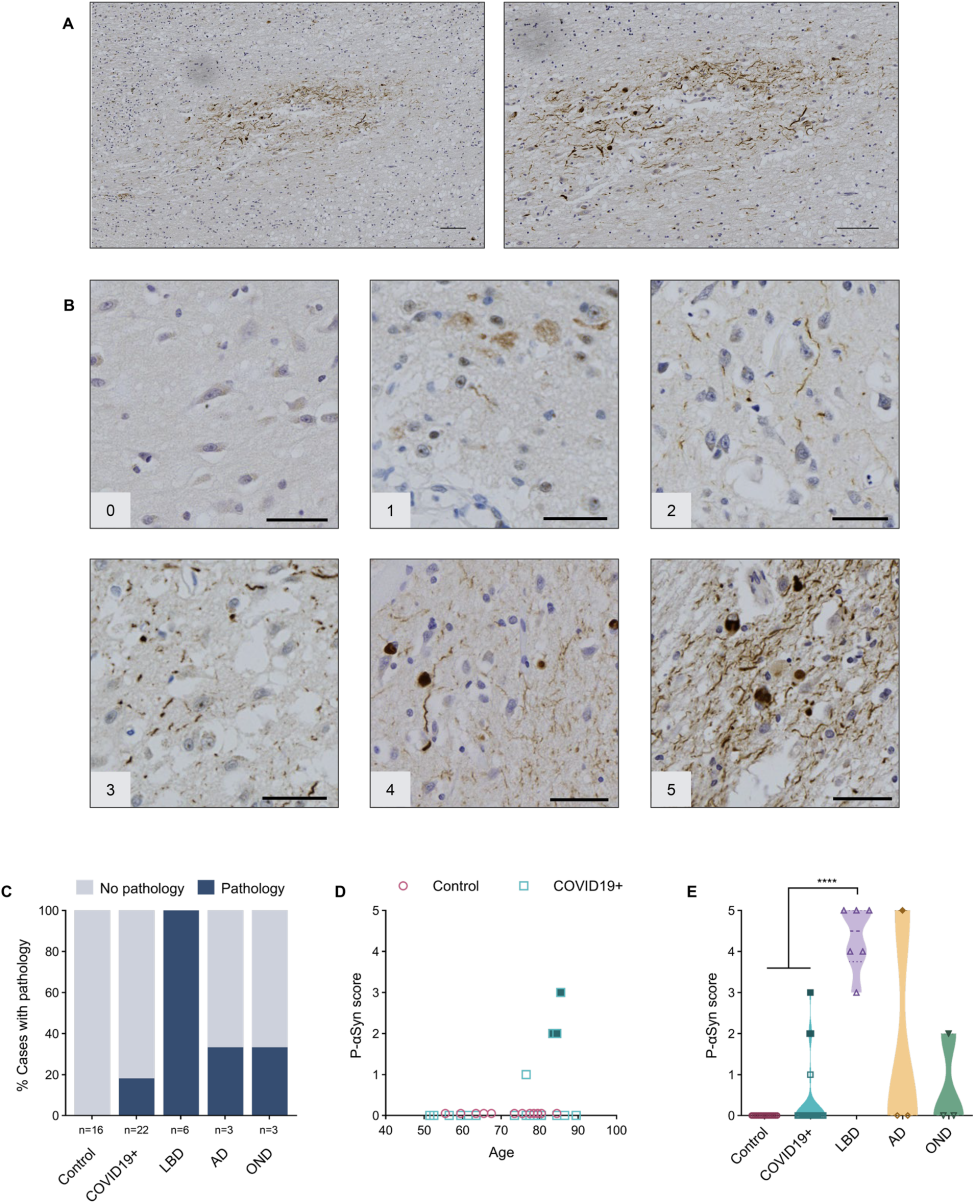
Anti-phosphorylated α-synuclein reactivity in the anterior olfactory nucleus.** A** Example of immunohistochemical staining for p-αSyn in the human olfactory bulb, highlighting the AON from a person with Parkinson disease and related dementia [case #39]. Scale bars represent 100 µM. **B** Representative images of semi-quantitative scoring of pathology, ranging from 0 to 5, in the AON. Scale bars represent 50 µM. **C** Percentage of cases in each group that have a pathology score of 1 or higher. **D** Correlation between age and p-αSyn pathology scores in the control group (HCO and NCO combined) and COVID19 + cases. **E** Distribution of pathology scores for each group. Filled blue squares in D and E indicate COVID19 + cases suspected of having incidental LBD at autopsy; filled dark yellow diamond in E indicates AD case diagnosed with mixed pathology at autopsy, and filled green triangle indicates MSA case. Significance was determined using Kruskal–Wallis test with Dunn's post-hoc (E), where **** indicates p ≤ 0.0001. Abbreviations for disease groups as in Fig. 1

Phosphorylated αSyn (p-αSyn)-positive pathology was found to be restricted to the AON (Fig. 3A). There, immunostaining was also scored on a semi-quantitative scale (as above) with representative images shown in Fig. 3B. Not surprisingly, p-αSyn pathology was detected within the AON of all LBD cases (Fig. 3C). Lewy neurites (LNs) and smaller, granular neuropil inclusions were found in several AON, but no definite, typical spherical Lewy bodies (LBs) within neuronal perikarya were observed. In the MSA case, abundant, pathognomonic glial cytoplasmic inclusions of oligodendrocytes were identified throughout the OB and OT (Additional file 1: Fig. S1). Typical p-αSyn pathology was detected in four of 22 (18%) COVID19 + cases. Three subjects had no documented history of clinical signs of parkinsonism, whereas one was suspected to have parkinsonism (without a formal neurological diagnosis) (Fig. 3C). In our series, anti-p-αSyn reactivity did not correlate with age (Fig. 3D). Importantly, three of four anti-p-αSyn-positive COVID19 + subjects showed evidence of ‘incidental LBD’ in the form of LBs and LNs in the Substantia nigra and dorsal motor nucleus of the vagus nerve. A fourth p-αSyn-positive COVID19 + case showed only a single reactive neurite in the OB, thus being assigned a score of 1. As expected, LBD cases were found to have a significantly higher average pathology score (4.3) than the control groups and COVID19 + cases (Fig. 3E). One AD case with positive p-aSyn pathology staining was diagnosed with mixed pathology (AD plus LBD), when assessing the entire brain (Fig. 3C; Additional file 1: Table S1). Immunohistochemical analyses using the anti-αSyn antibody, LB509, revealed similar results when compared to those using anti-p-aSyn (Additional file 1: Fig. S2).

**Fig. 4:**
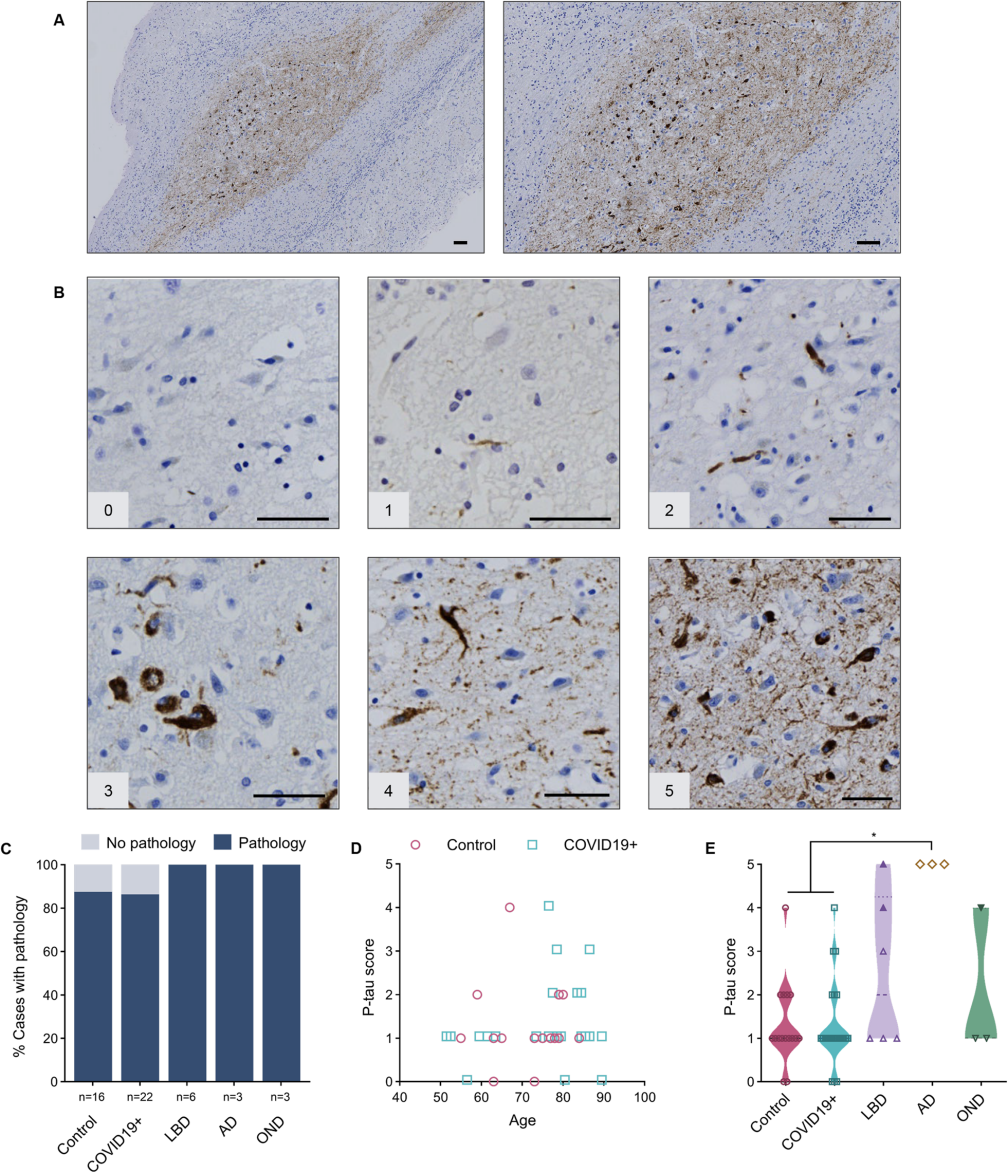
Anti-phosphorylated tau reactivity in the anterior olfactory nucleus. **A** Example of immunohistochemical staining for p-tau in the human olfactory bulb, concentrated in the AON, from a person with Alzheimer disease [case #47]. Scale bar represents 100 µM. **B** Representative images of semi-quantitative scoring of pathology, ranging from 0 to 5, in the AON. Scale bar represents 50 µM. **C** Percentage of cases in each group that have a pathology score of 1 or higher. **D** Correlation between age and p-tau scores for the control (HCO and NCO) and COVID19 + groups. **E** Distribution of tau pathology score for each group; filled purple triangles indicate LBD cases diagnosed with mixed pathology at autopsy. Filled green triangle indicates MSA case. Significance was determined using Kruskal–Wallis test with Dunn's post-hoc, where * indicates p ≤ 0.05. Abbreviations for disease groups as in Fig. 1

Phosphorylated tau (p-tau) inclusions were detected by the AT8 antibody in 90% of the cases and were confined to the AON (Fig. 4A). These tau-immunoreactive aggregates included dystrophic neurites as well as neurofibrillary tangles. Representative images corresponding to the semi-quantitative scale used for scoring (as above) are shown in Fig. 4B. In this series, there was no correlation between the severity of p-tau pathology and the age of neurologically healthy cases (Fig. 4D). As expected, AD cases had the highest average pathology score (5.0) among all groups; it was significantly higher than reactivity seen in the control and COVID19 + groups (Fig. 4E).

**Fig. 5:**
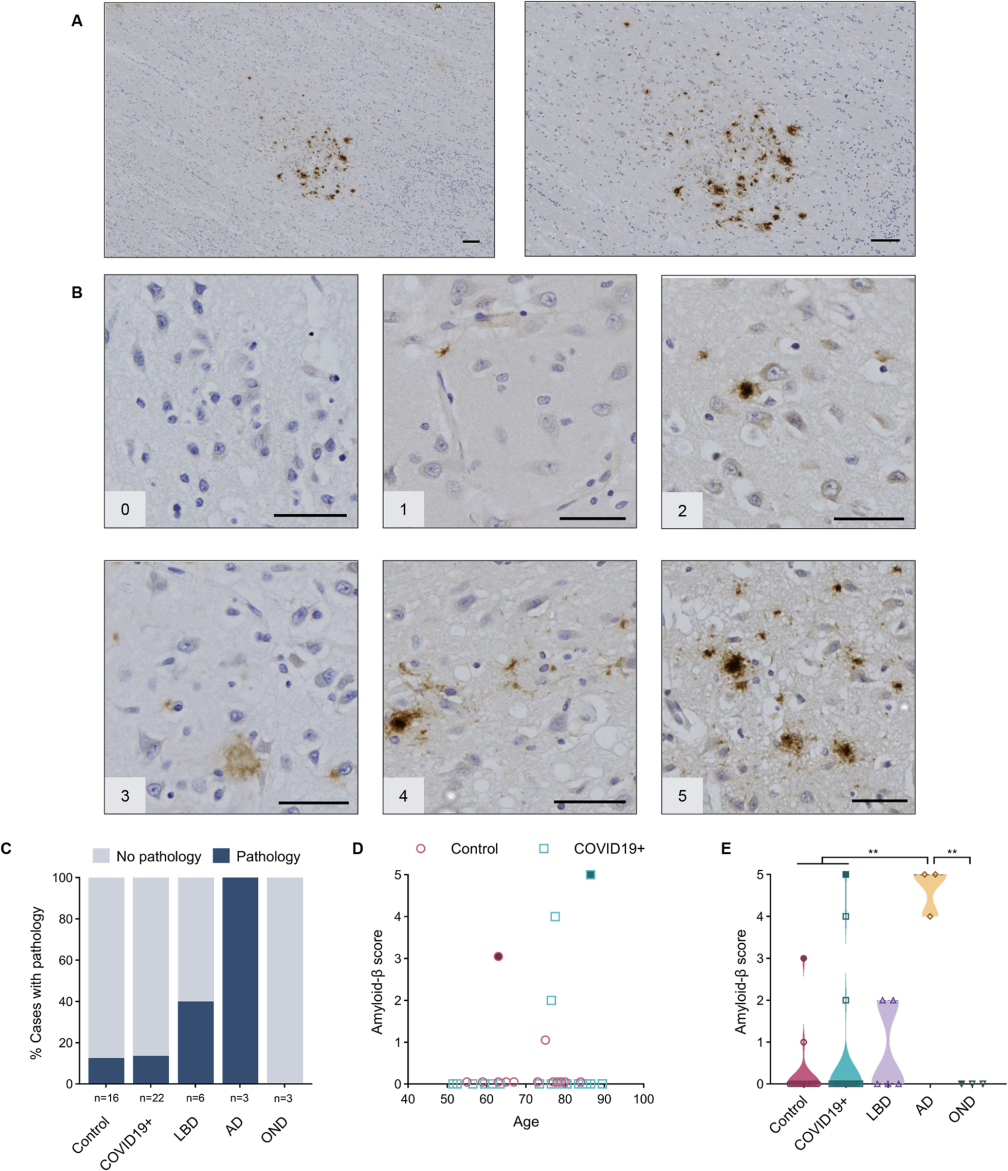
Anti-amyloid-β peptide reactivity in the anterior olfactory nucleus.** A** Example of immunohistochemical staining for amyloid-β peptide in human olfactory bulb, concentrated in the AON, from a person with Alzheimer disease [case #46]. Scale bar represents 100 µM. **B** Representative images of semi-quantitative scoring of pathology, ranging from 0 to 5, in the AON. Scale bar represents 50 µM. **C** Percentage of cases in each group that have a pathology score of 1 or more. **D** Correlation between age and amyloid-β peptide scores in the control (HCO and NCO) and COVID19 + groups. Subject highlighted in blue is suspected to have Alzheimer disease (AD) based on neuropathological findings in the temporal lobes. **E** Distribution of pathology score for each group. Filled maroon circle (in D, E) indicates a control subject with amyloid-β-related angiitis; filled blue square indicates COVID19 + case suspected of having AD. Significance was determined using Kruskal–Wallis test with Dunn's post-hoc, where ** indicates p ≤ 0.01. Abbreviations for disease groups as in Fig. 1

When detected, amyloid-β peptide-specific pathology was found to be restricted to the AON (Fig. 5A), with representative images shown in Fig. 5B. As expected, all cases in the AD group were found to have amyloid-β pathology. A small percentage of control and COVID19 + cases were found to have amyloid-β plaques in the AON, while other neurodegenerative cases did not show any detectable amyloid-β-positive pathology in their OB or OT (Fig. 5C). We saw no correlation between the degree of amyloid-β peptide burden and age in control or COVID19 + cases (Fig. 5D). One NCO case (grouped here together with HCO) with amyloid-β-related angiitis generated an amyloid-β score of 3. The COVID19 + brain that contained the highest amyloid-β peptide load showed AD neuropathologic changes on *post mortem* examination of other brain regions despite the absence of a documented clinical history of dementia. When looked at as a group, AD cases had a significantly higher average score for amyloid-β deposition (4.7), as expected, than corresponding tissues from controls, COVID19 + and OND subjects (Fig. 5E).

**Fig. 6:**
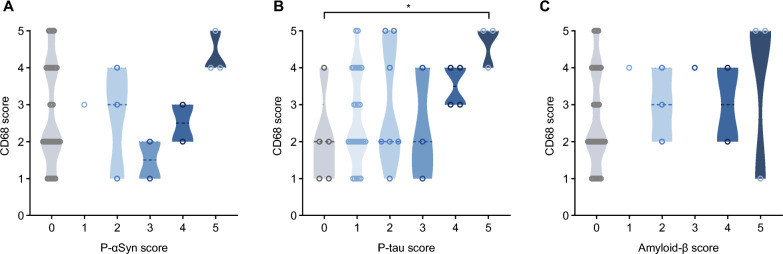
Correlation studies between microglial activation and neuropathological findings in the anterior olfactory nucleus. Violin plots show the relationships between scores for anti-CD68 immunoreactivity and three neurodegeneration-linked proteins in the AON, namely phosphorylated α-synuclein (**A**), phosphorylated tau (**B**) and amyloid-β peptide (**C**). Significance was determined using Kruskal–Wallis test with Dunn's post-hoc, using a score of 0 (indicating absent pathology) for comparison, where * indicates p ≤ 0.05

Lastly, we sought to correlate staining for neurodegenerative proteins with the degree of CD68-immunoreactivity (Fig. 6A–C). When comparing the cases with a pathology score of 1 or above to those with no pathology (score 0), p-tau cases with a score of 5 had significantly higher CD68 reactivity (Fig. 6B). Individual trends but no significant differences were seen when correlating anti-CD68 immunoreactivity scores with the degree of synucleinopathy or amyloid-β peptide amyloidosis (Fig. 6A, C).

Individual scores for the immunostaining with each antibody for each of the 50 autopsy cases are shown in Table 2, with additional details summarized in Additional file 1: Tables S3, S4, S5, S6 and S7.

**Table 2:**
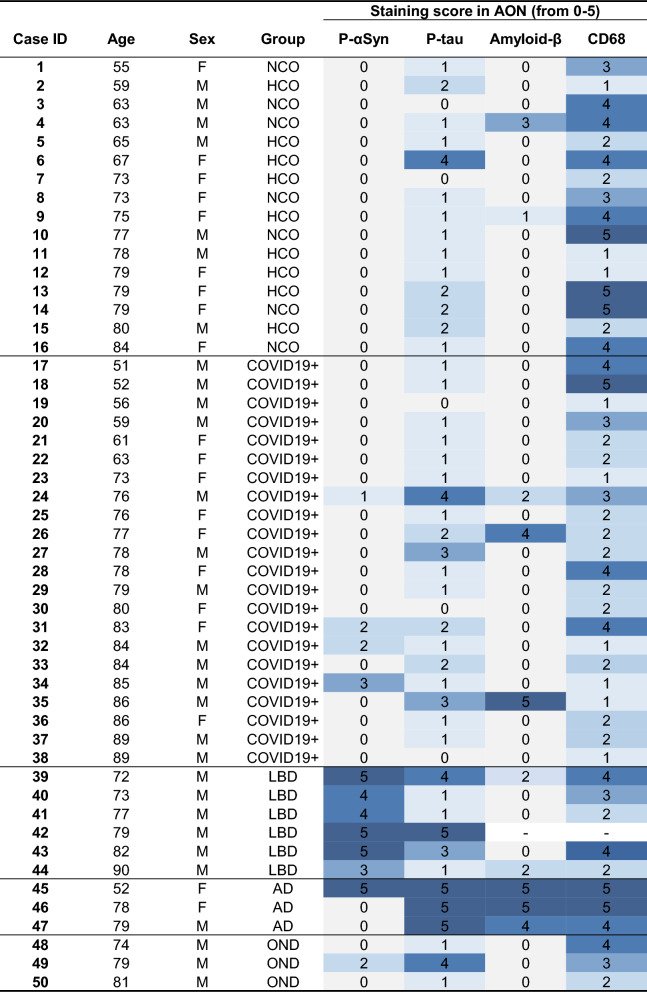
Heat map of all staining scores for each case in this study

Characteristics listed include: age (in years); sex (F, female; M, male); diagnostic group (HCO, healthy control; NCO, neurological control; LBD, Lewy body diseases; AD, Alzheimer disease; OND, other neurological disease); and pathology scores in the AON (anterior olfactory nucleus); for each marker, range 0 to 5

## Discussion

Previous authors have pointed at a possible association between SARS-CoV-2 infection and neurodegeneration, as identified by a rise in the incidence of cognitive decline and parkinsonism in survivors [29, 34–36]. A recent imaging study of COVID19 + subjects, including those with mild disease, revealed reductions in grey matter thickness in olfaction-related structures as well as other regions functionally connected to the olfactory cortex [16]. In contrast, other investigators [37, 38] reported increases in gray matter volumes in distinct anatomical regions. These divergent results likely reflect substantial complexity in the relationship between pathogenic SARS-CoV-2 variants, genetic susceptibility of the host, mechanisms of disease, and the period that has passed between actual infection and the assessment of outcomes.

Our study was limited to subacute COVID-19 infections, with an average hospitalization length of two weeks prior to the patients’ passing (range, 1–49 days). Within this context of a subacute illness, we did not find any evidence for a relationship between the history of a recent COVID-19-related illness, which had warranted hospital admission and was ultimately fatal, and features of αSyn pathology in the OB or AON, using immunohistochemical staining with two well-characterized, specific antibodies as a readout. Although we detected αSyn aggregates within the AON in four of 22 (18%) of the neurologically intact COVID19 + group, three of these subjects showed typical Lewy pathology in the brainstem, with only one of those having had suspected parkinsonism as per the medical record. It is generally thought that these hallmark findings of PD are generated over a decade (or more) [39], whereas the course of COVID-19 illness that led to death in our study subjects was on average two weeks following hospitalization (Additional file 1: Table S1). Therefore, we speculate that the observed pathology in these cases reflected pre-existing LBD that occurred independently of SARS-CoV-2 infection. In this respect, our results are compatible with those of Blanco-Palmero and co-workers, who observed no significant difference in total αSyn levels in the cerebrospinal fluid or serum among COVID-19 patients versus HCO subjects [40].

However, our findings do not exclude an effect by the RNA virus infection on subtle αSyn misprocessing within the olfactory system, as the duration of illness was short and may have been insufficient for immunohistochemically detectable, fibrillar αSyn aggregates to form. An implication of this caveat is that in the future more sensitive approaches for the detection of soluble oligomers, of post-translationally modified conformers, of in vitro seeding-competent species or of alternatively spliced mRNA transcripts could reveal such an association, including in the early stages after COVID-19 infection.

Previous *post mortem* analyses of COVID-19 patients had revealed local inflammation associated with axonal pathology and microvascular changes in the OB and OT [15] as well as the finding of increased immune activation and microglial nodules, as reported for example by Schwabenland et al. [41]. In our study, the absence of significant microglial activation in COVID19 + subjects relative to controls, as judged by using CD68 immunoreactivity as a readout, is consistent with findings by Matschke et al. and Seranno et al. [11, 42]. There, changes in the OB of COVID-19 cases had been identified, including at the transcriptional level due to deafferentation [42, 43]. In our case series and regarding the evidence of *bona fide* αSyn aggregates, this relative paucity of reactivity within the OB and OT could be related not only to the short duration of illness, but also to a transient nature of infection within the nasal cavity, or to the lack of actual tissue invasion by virions. Conversely, one could speculate that less visible inflammation reflects a relatively deficient immune response by older subjects (and rendering them more susceptible to COVID-19 infection in general). Along these lines, we found that CD68 scores in the rostral olfactory circuitry were negatively associated with age in our COVID19 + cases, possibly reflecting a decline in anti-viral responses. Studies are underway to examine the degree of inflammation in the piriform cortex, to which the olfactory tracts project.

One shortcoming of our study is that no objective assessment of olfactory function had been performed in any of the participants that subsequently died within the hospital, in large part due to the acuity and severity of respiratory distress that had warranted admission. In a parallel effort, we are currently analyzing serial sections of the intact nasal cavity, including olfactory and respiratory epithelia, from all 50 study participants, where we will revisit the rate of detection of SARS-CoV-2 nucleocapsid proteins. When probing for the presence of viral proteins in OB and OT sections from three, PCR-confirmed COVID19 + cases in our series, we detected no specific evidence of a viral infection.

Although we were unable to procure evidence for a direct relationship between COVID-19 infection and olfactory αSyn aggregation following the short duration of illness studied herein, our study revealed interesting findings of relevance to olfactory dysfunction in neurodegeneration and perhaps to ageing as well. For example, our demonstration that the overall severity of αSyn as well as amyloid-β and tau pathology within the OB and AON ranked highest in LBD and AD cases, respectively, is consistent with the frequent association of hyposmia with both disorders. Conversely, the relative paucity of αSyn and tau pathology within AON neurons from our MSA and PSP subjects, respectively, correlates with the absence of a loss in sense of smell as a cardinal clinical manifestation in these diseases [44].

The existence of tau and αSyn co-pathology in almost all of the synucleinopathy cases in our series suggests that the molecular mechanisms, which underlie the frequent co-existence of these changes in the brains of AD and LBD patients, are also operative in the intracranial olfactory circuitry.

Our rationale for including subjects with AD derived from evidence that SARS-CoV-2 infection may facilitate cognitive decline [29] and may disrupt both amyloid-β and tau homeostasis, leading to tau hyperphosphorylation, as suggested in part by elevated plasma concentrations of both total and phosphorylated tau in living subjects [45–48]. We detected no strong evidence yet to link COVID-19 infection to β-amyloidosis in the rostral olfactory circuitry. However, three COVID19 + cases (13.6%) compared to a single control individual (6.25%) were found to have amyloid-β pathology in the OB and OT. Although the numbers of cases in each diagnostic group were too small to draw definitive conclusions, this result is intriguing considering studies that showed SARS-CoV-2, herpetiform DNA viruses and select bacteria can alter amyloid-β homeostasis and modify neuropathological outcomes [45, 49].

We detected no evidence for a relationship between COVID-19 illness and p-tau deposition in the OB or AON at autopsy. Consistent with recent findings by Tremblay and colleagues [50], 90% of our cohort, including control participants, displayed at least some degree of tau pathology. This is perhaps not surprising given that the process of tau hyperphosphorylation and aggregation is not confined to primary neurodegenerative diseases. Indeed, changes in tau metabolism are increasingly recognized as sequelae of diverse insults to the brain, including seizures, trauma, viral infections and autoimmune disease [51, 52], and as a part of physiological ageing [50]. In this context, the prevalence of tau pathology in the OB and AON across our entire cohort may represent a morphological surrogate for a variety of insults, including pathogen-induced inflammation, sustained by individuals throughout their lifetime. Perhaps consistent with this, tauopathy cases in our series showed the highest anti-CD68 reactivity scores (Fig. 6B) although here too group sizes were underpowered to allow definitive conclusions. Moreover, although p-tau aggregation and microglial/histiocytic responses could be functionally related, it is not possible to ascribe temporal precedence to one or the other due to the cross-sectional nature of our study.

In conclusion, we found no evidence that a subacute, fatal SARS-CoV-2 infection induces typical αSyn aggregates in the OB or AON of human subjects under the microscopic conditions examined. However, our study has limitations and further investigations addressing these are required prior to excluding a role for this RNA virus in triggering neurodegeneration within the olfactory system. The most critical of these is the discrepancy between the interval of COVID-19 illness, which in our cohort was short, and the time required for neurodegenerative changes to be detectable by traditional immunohistochemistry. Related to this, the subjects in our cohort were all above 50 years of age, thereby complicating the attribution of neurodegenerative changes to SARS-CoV-2 alone versus those related to ageing, including those clinically not yet detected. Future studies should include more subjects, including younger individuals, those with a longer duration of viral illness and those with neurological signs as a result of their infectious illness. We will supplement histological analyses with more sensitive, biochemically based techniques and quantification of transcriptional changes to detect pathologically relevant structural and molecular alterations in the proteins of interest. In our prospectively collected cohort, there were no female subjects in our LBD and OND groups, precluding analysis of sex-related differences in these subsets. We were also underpowered in our neurodegenerative disease case numbers, which could have contributed to the negative findings here, including the lack of evidence for increased microglial activation of the OB/OT in the COVID19 + group (Fig. 2C). Further, because this *post mortem* study was cross-sectional, it precluded analyses of any temporal relationship(s) among the changes observed.

To address any long-term sequelae *versus* short-term effects of RNA virus infections of the upper respiratory tract on brain health, such as pertaining to αSyn, tau and amyloid-β peptide homeostasis, we have recently begun to conduct experiments in mice, including those that carry PD-linked allelic variants [17, 53]. These ongoing studies in animal models include the inoculation of the nasal cavity with a mouse-adapted strain of human SARS-CoV-2 [33] to monitor its potential aggregation effects on neurodegeneration-linked proteins. As mentioned, our investigation here was confined to the OB/OT and AON. Autopsy studies interrogating the impact of SARS-CoV-2 infection in humans on αSyn metabolism (and inflammation-related signaling) at a more rostral site in the olfactory epithelium as well as more caudally in the piriform cortex are currently ongoing. We anticipate that these will add to an ultimately anatomically more complete assessment of this potentially important environment-brain interaction.

## Supplementary Information

**Additional file 1**. Figure S1: Phosphorylated α-synuclein pathology in the anterior olfactory nucleus of a multisystem atrophy case. Example of phosphorylated α-synuclein pathology shown at low and high magnifications depicting glial cytoplasmic inclusions in oligodendrocytes of the anterior olfactory nucleus in the olfactory bulb of an individual with multisystem atrophy (type-P; case #49). Scale bars represent 100µM. Figure S2: LB509-mediated α-synuclein reactivity in the anterior olfactory nucleus. A) Percentage of cases in each group that have a pathology score of 1 or higher. B) Correlation between age and LB509-based α-synuclein pathology scores in the control (HCO and NCO) and COVID19+ groups. C) Distribution of LB509 pathology scores for each group. Filled blue squares are subjects suspected of having incidental LBD; filled dark yellow diamond reflects a subject with mixed pathology. D) Relationship between anti-CD68 scores and LB509-based α-synuclein pathology scores in the AON of all subjects. Significance was determined using Kruskal-Wallis test with Dunn's post-hoc, where ** denotes p≤0.01 and **** denotes p≤0.0001. Abbreviations for disease groups as in Fig. 1. Table S1: Detailed characteristics of all subjects examined at autopsy. Characteristics listed include age (in years); sex (F, female; M, male); diagnostic group (HCO, healthy control; NCO, neurological control; LBD, Lewy body disorders; AD, Alzheimer disease; OND, other neurological disease); pathology diagnosis at autopsy; ventilation; clinical cause of death; number of days in hospital; site of tissue collection; tissue analyzed; post mortem interval (PMI; in hours); hippocampal (hippocamp.) staining; α-synuclein staining in the Substantia nigra (SN) and dorsal motor nucleus of the vagus nerve (DNV). * Indicate cases with an inflammatory condition. # Indicate cases with mixed pathology. Abbreviations: AD, Alzheimer disease; ADNC, Alzheimer disease neuropathologic change. AxBxCx scoring of AD-linked neuropathology in the hippocampus was applied as per Hyman BT et al., 2012 Alzheimer’s Dementia Jan;8(1):1-13; AML, acute myeloid leukemia; DAD, diffuse alveolar disease; ECMO, extracorporeal membrane oxygenation; LBD, Lewy body diseases; DLB, dementia with Lewy bodies; PSP, progressive supranuclear palsy; FTLD-TDP, frontotemporal lobar degeneration-Tar DNA-binding protein; MSA-P, multisystem atrophy-parkinsonian type (P); N, no; NA, not applicable or not available; Y, yes. Table S2: Resources and reagents used in study. Table S3: Detailed results for phosphorylated α-synuclein staining of each case. Abbreviations for disease groups as in Table S1. N, no; Y, yes. Table S4: Detailed results for phosphorylated tau staining of each case. Abbreviations for disease groups as in Table S1. Table S5: Detailed results for amyloid-β peptide staining of each case. Abbreviations for disease groups as in Table S1. AxBxCx scoring of Alzheimer disease-linked neuropathology in the hippocampus was applied as per Hyman BT et al., 2012 Alzheimer’s Dementia Jan;8(1):1-13; NA, not applicable or not available; N, no; Y, yes. Table S6: Detailed results for CD68 staining of each case. Abbreviations for disease groups as in Table S1. Table S7: Detailed results for LB509-based α-synuclein staining of each case. Abbreviations for disease groups as in Table S1.

## Data Availability

Original data associated with this study are available in the main text and supplementary figures and tables, and can be downloaded at doi.org/10.5281/zenodo.10776590. Additional data will be made available upon request.
