# Correction: Neuropathological assessment of the olfactory bulb and tract in individuals with COVID-19

**DOI:** 10.1186/s40478-024-01843-7

**Published:** 2024-09-19

**Authors:** Nathalie A. Lengacher, Julianna J. Tomlinson, Ann‑Kristin Jochum, Jonas Franz, Omar Hasan Ali, Lukas Flatz, Wolfram Jochum, Josef Penninger, aSCENT-PD Investigators, Christine Stadelmann, John M. Woulfe, Michael G. Schlossmacher

**Affiliations:** 1 Neuroscience Program, Ottawa Hospital Research Institute, Ottawa, ON Canada; 2 Institute of Pathology, Kantonsspital St. Gallen, St. Gallen, Switzerland; 3 Institute of Immunobiology, Kantonsspital St. Gallen, St. Gallen, Switzerland; 4 Neuropathology Institute, University of Goettingen Medical Centre, Goettingen, Germany; 5 Department of Life Sciences, University of British Columbia, Vancouver, BC Canada; 6 Department of Pathology and Laboratory Medicine, The Ottawa Hospital, Ottawa, ON Canada; 7 Department of Dermatology, University Hospital Tubingen, Tubingen, Germany; 8 Division of Neurology, Department of Medicine, The Ottawa Hospital, Ottawa, ON Canada; 9 Aligning Science Across Parkinson’s (ASAP) Collaborative Research Network, Chevy Chase, MD 20815 USA

**Correction: Acta Neuropathologica Communications (2024) 12:70** 10.1186/s40478-024-01761-8

Following publication of the original article [1], in Fig. 3, B panel image “1” is incorrect. The incorrect section of the Fig. 3B and corrected version of full Fig. 3 is given below.

Incorrect Fig. 3, Panel B, Image 1:
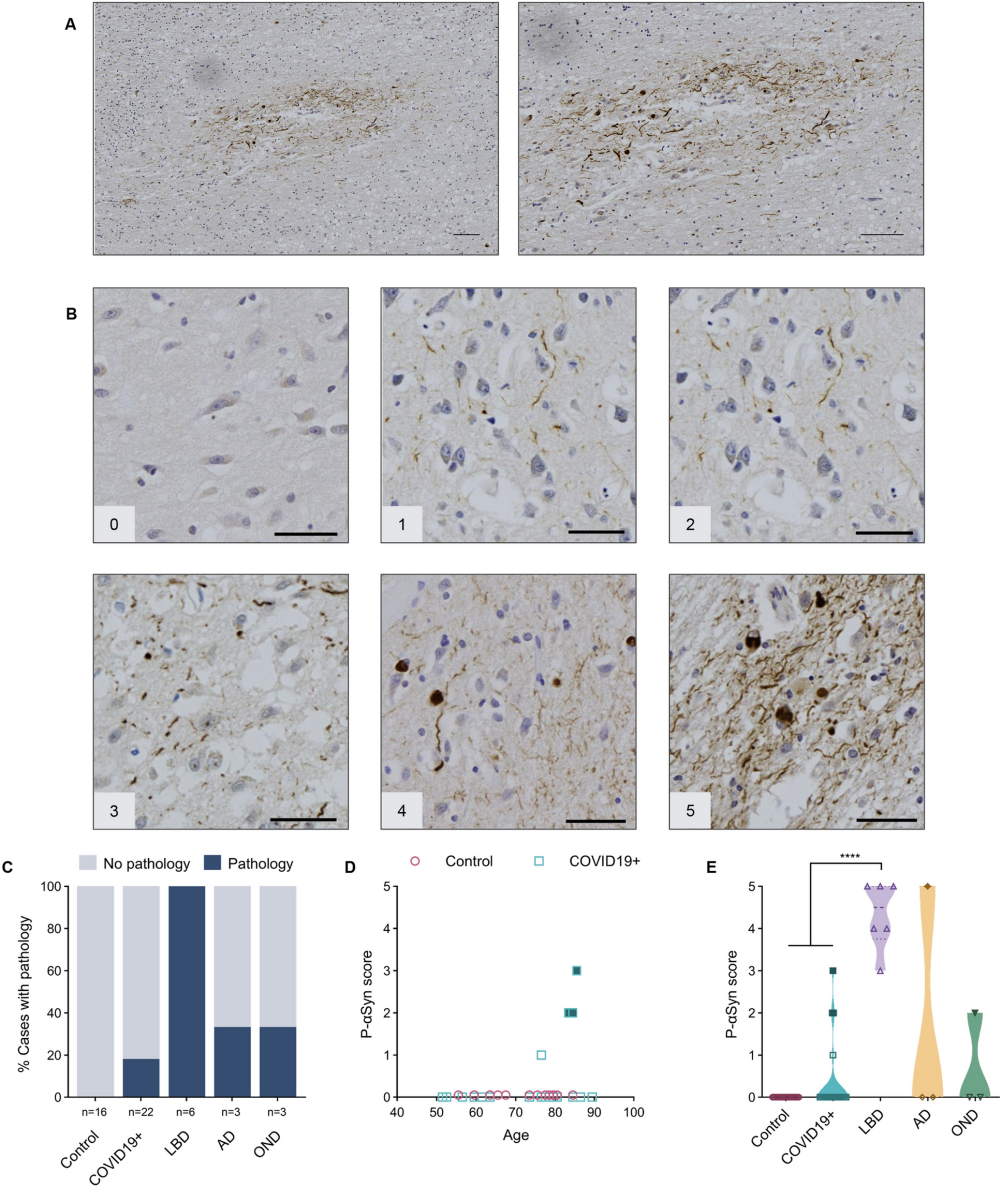


Figure 3 and caption.

**Fig. 3:**
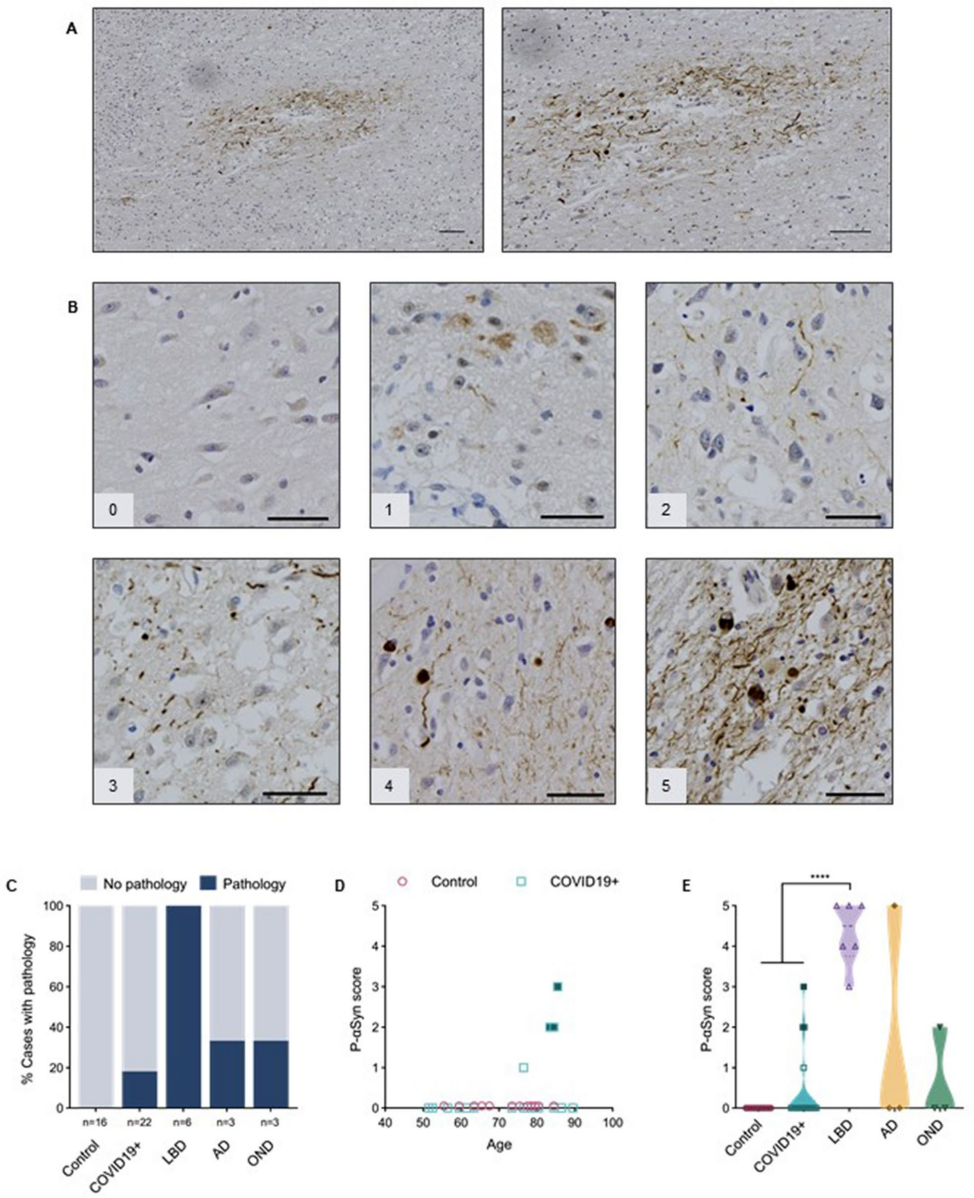
Anti-phosphorylated α-synuclein reactivity in the anterior olfactory nucleus. **A** Example of immunohistochemical staining for p-αSyn in the human olfactory bulb, highlighting the AON from a person with Parkinson disease and related dementia [case #39]. Scale bars represent 100 μM. **B** Representative images of semi-quantitative scoring of pathology, ranging from 0 to 5, in the AON. Scale bars represent 50 μM. **C** Percentage of cases in each group that have a pathology score of 1 or higher. **D** Correlation between age and p-αSyn pathology scores in the control group (HCO and NCO combined) and COVID19 + cases. **E** Distribution of pathology scores for each group. Filled blue squares in **D** and **E** indicate COVID19 + cases suspected of having incidental LBD at autopsy; filled dark yellow diamond in **E** indicates AD case diagnosed with mixed pathology at autopsy, and filled green triangle indicates MSA case. Significance was determined using Kruskal–Wallis test with Dunn’s post-hoc (**E**), where **** indicates *p* ≤ 0.0001. Abbreviations for disease groups as in Fig. 1

The original article has been corrected.
