# Supplementary material for: Neuropathological assessment of the olfactory bulb and tract in individuals with COVID-19
Source: Acta Neuropathol Commun. 2024 May 3;12:70. doi: 10.1186/s40478-024-01761-8 (PMC11067107; doi:10.1186/s40478-024-01761-8)
Supplement: Supplementary file 1 — Additional file 1. Figure S1: Phosphorylated α-synuclein pathology in the anterior olfactory nucleus of a multisystem atrophy case. Example of phosphorylated α-synuclein pathology shown at low and high magnifications depicting glial cytoplasmic inclusions in oligodendrocytes of the anterior olfactory nucleus in the olfactory bulb of an individual with multisystem atrophy (type-P; case #49). Scale bars represent 100µM. Figure S2: LB509-mediated α-synuclein reactivity in the anterior olfactory nucleus. A) Percentage of cases in each group that have a pathology score of 1 or higher. B) Correlation between age and LB509-based α-synuclein pathology scores in the control (HCO and NCO) and COVID19+ groups. C) Distribution of LB509 pathology scores for each group. Filled blue squares are subjects suspected of having incidental LBD; filled dark yellow diamond reflects a subject with mixed pathology. D) Relationship between anti-CD68 scores and LB509-based α-synuclein pathology scores in the AON of all subjects. Significance was determined using Kruskal-Wallis test with Dunn's post-hoc, where ** denotes p≤0.01 and **** denotes p≤0.0001. Abbreviations for disease groups as in Fig. 1. Table S1: Detailed characteristics of all subjects examined at autopsy. Characteristics listed include age (in years); sex (F, female; M, male); diagnostic group (HCO, healthy control; NCO, neurological control; LBD, Lewy body disorders; AD, Alzheimer disease; OND, other neurological disease); pathology diagnosis at autopsy; ventilation; clinical cause of death; number of days in hospital; site of tissue collection; tissue analyzed; post mortem interval (PMI; in hours); hippocampal (hippocamp.) staining; α-synuclein staining in the Substantia nigra (SN) and dorsal motor nucleus of the vagus nerve (DNV). * Indicate cases with an inflammatory condition. # Indicate cases with mixed pathology. Abbreviations: AD, Alzheimer disease; ADNC, Alzheimer disease neuropathologic change. AxBxCx scoring of AD-li [file 40478_2024_1761_MOESM1_ESM.pptx]

## Slide 1
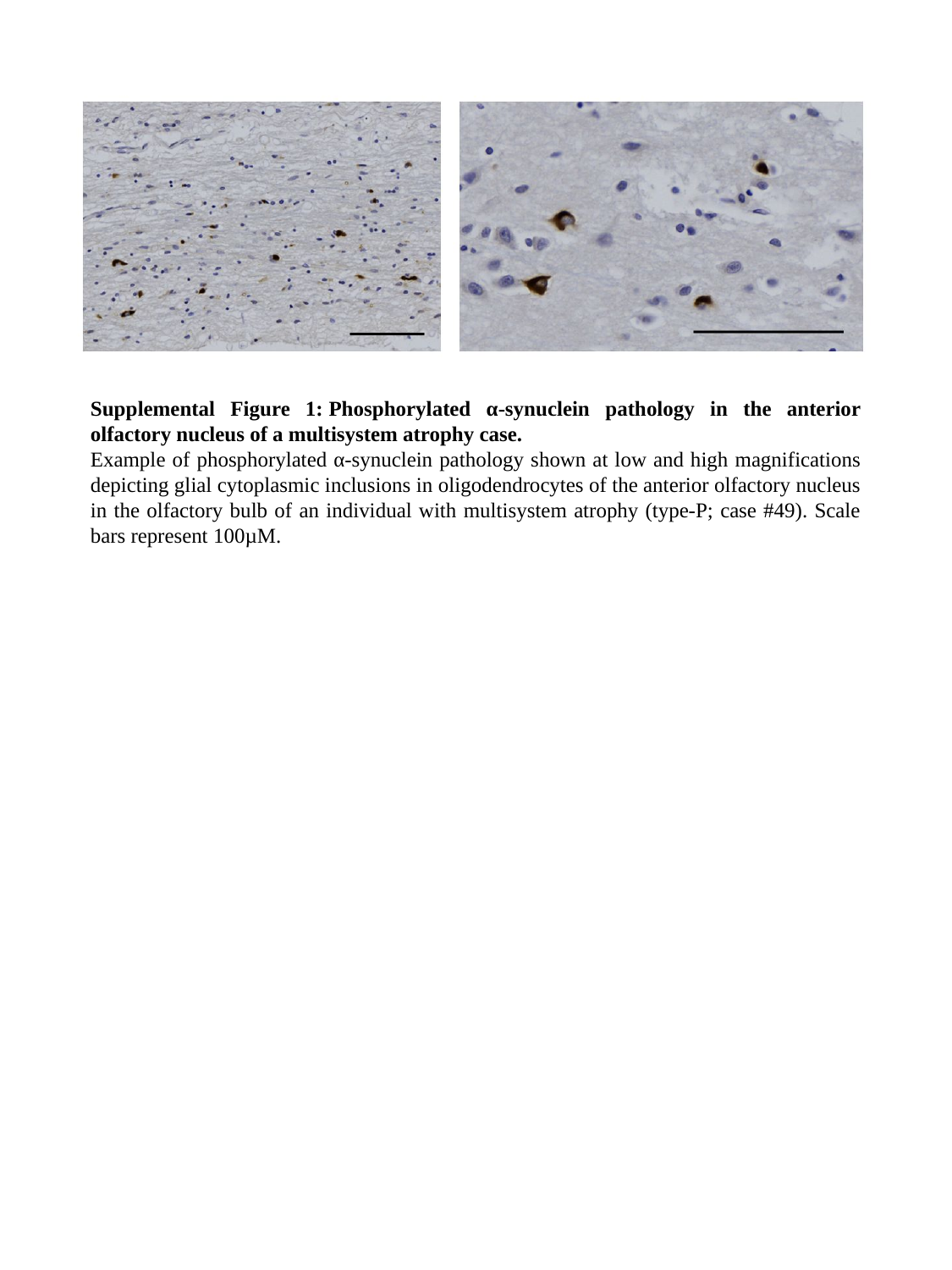

Supplemental Figure 1: Phosphorylated α-synuclein pathology in the anterior olfactory nucleus of a multisystem atrophy case.
Example of phosphorylated α-synuclein pathology shown at low and high magnifications depicting glial cytoplasmic inclusions in oligodendrocytes of the anterior olfactory nucleus in the olfactory bulb of an individual with multisystem atrophy (type-P; case #49). Scale bars represent 100µM.

## Slide 2
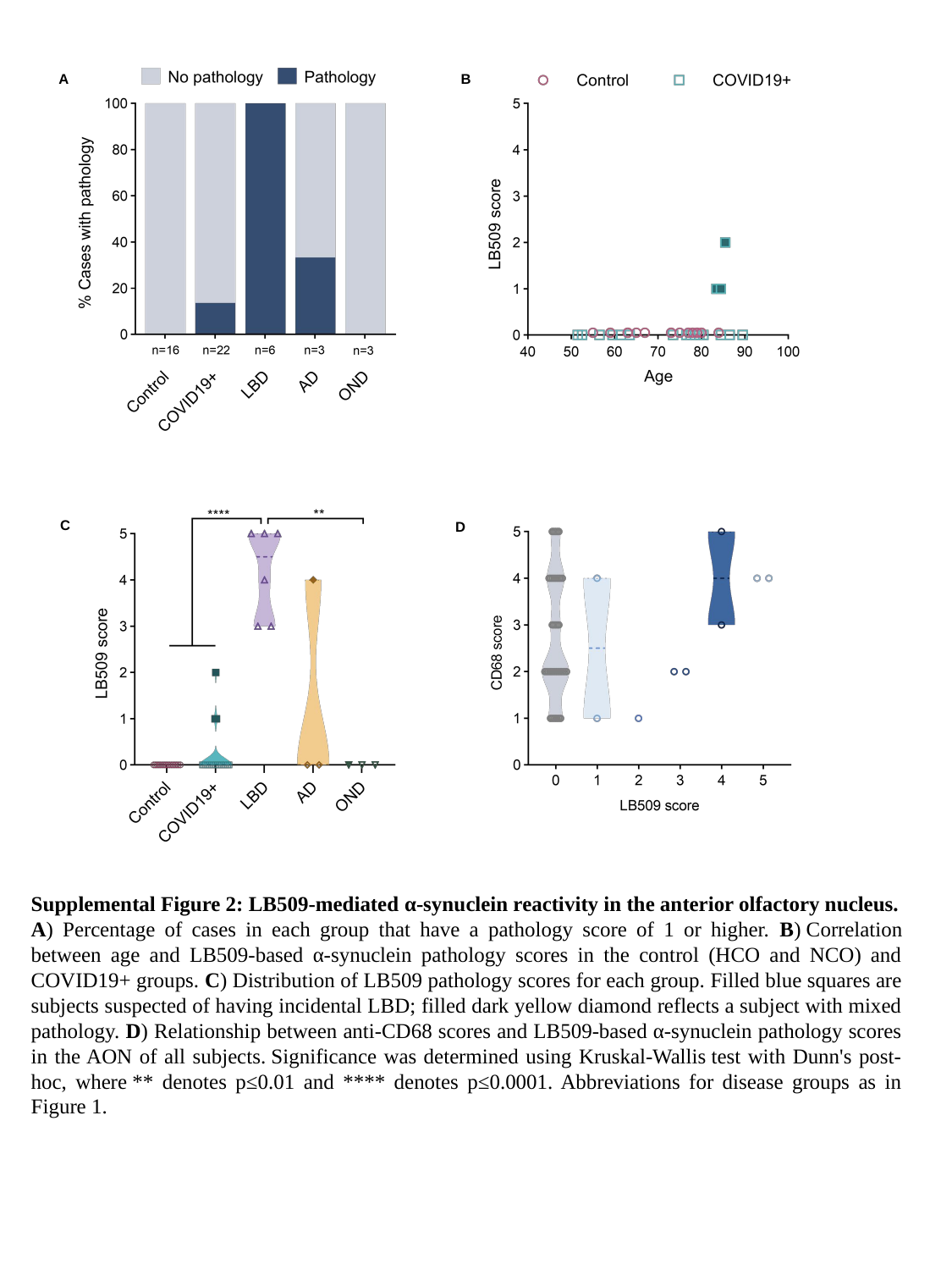

B
A
C
D
Supplemental Figure 2: LB509-mediated α-synuclein reactivity in the anterior olfactory nucleus.
A) Percentage of cases in each group that have a pathology score of 1 or higher. B) Correlation between age and LB509-based α-synuclein pathology scores in the control (HCO and NCO) and COVID19+ groups. C) Distribution of LB509 pathology scores for each group. Filled blue squares are subjects suspected of having incidental LBD; filled dark yellow diamond reflects a subject with mixed pathology. D) Relationship between anti-CD68 scores and LB509-based α-synuclein pathology scores in the AON of all subjects. Significance was determined using Kruskal-Wallis test with Dunn's post-hoc, where ** denotes p≤0.01 and **** denotes p≤0.0001. Abbreviations for disease groups as in Figure 1.

## Slide 3
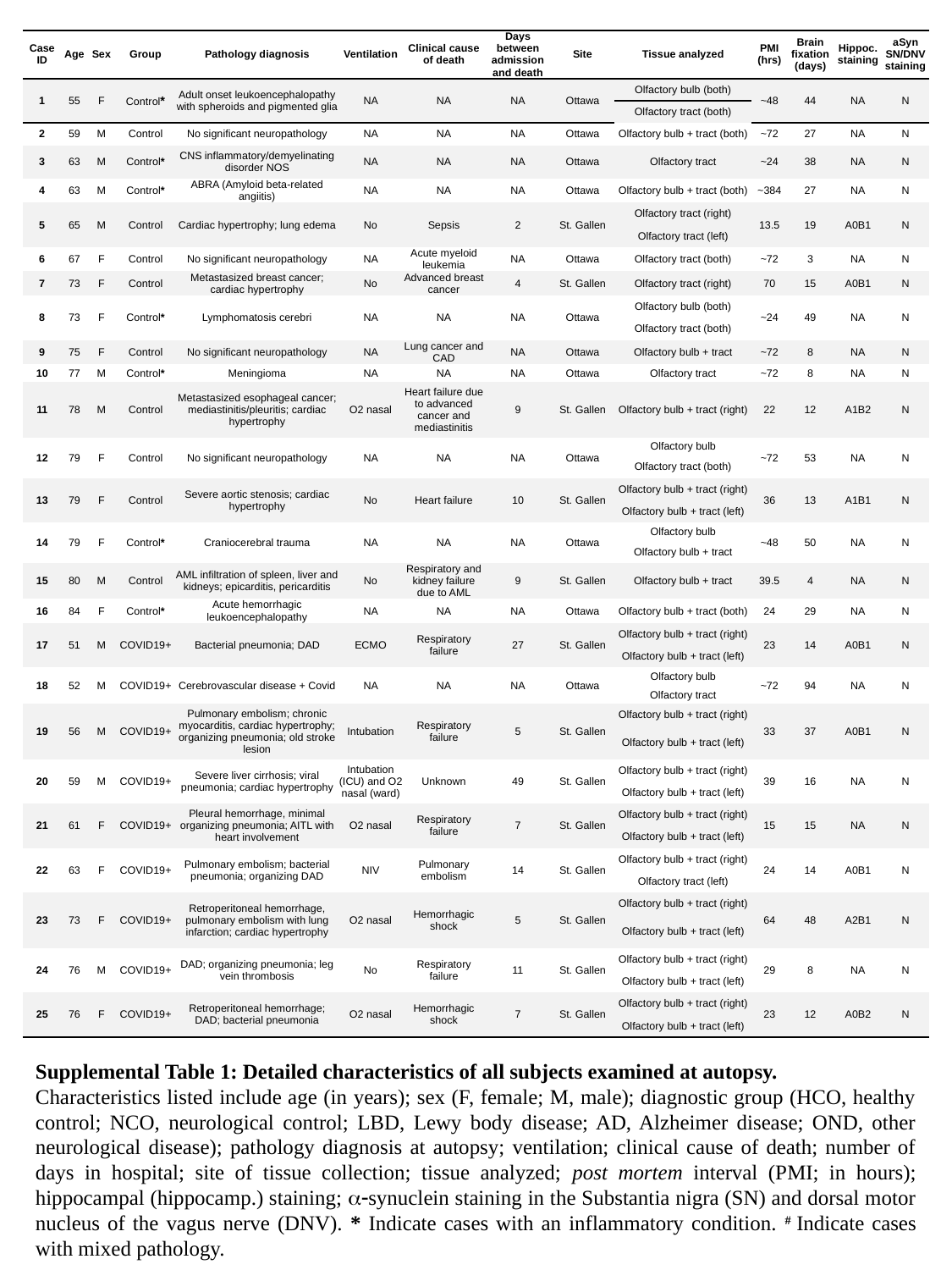

| Case ID | Age | Sex | Group | Pathology diagnosis | Ventilation | Clinical cause of death | Days between admission and death | Site | Tissue analyzed | PMI (hrs) | Brain fixation (days) | Hippoc. staining | aSyn SN/DNV staining |
| --- | --- | --- | --- | --- | --- | --- | --- | --- | --- | --- | --- | --- | --- |
| 1 | 55 | F | Control\* | Adult onset leukoencephalopathy with spheroids and pigmented glia | NA | NA | NA | Ottawa | Olfactory bulb (both) | ~48 | 44 | NA | N |
| | | | | | | | | | Olfactory tract (both) | | | | |
| 2 | 59 | M | Control | No significant neuropathology | NA | NA | NA | Ottawa | Olfactory bulb + tract (both) | ~72 | 27 | NA | N |
| 3 | 63 | M | Control\* | CNS inflammatory/demyelinating disorder NOS | NA | NA | NA | Ottawa | Olfactory tract | ~24 | 38 | NA | N |
| 4 | 63 | M | Control\* | ABRA (Amyloid beta-related angiitis) | NA | NA | NA | Ottawa | Olfactory bulb + tract (both) | ~384 | 27 | NA | N |
| 5 | 65 | M | Control | Cardiac hypertrophy; lung edema | No | Sepsis | 2 | St. Gallen | Olfactory tract (right) | 13.5 | 19 | A0B1 | N |
| | | | | | | | | | Olfactory tract (left) | | | | |
| 6 | 67 | F | Control | No significant neuropathology | NA | Acute myeloid leukemia | NA | Ottawa | Olfactory tract (both) | ~72 | 3 | NA | N |
| 7 | 73 | F | Control | Metastasized breast cancer; cardiac hypertrophy | No | Advanced breast cancer | 4 | St. Gallen | Olfactory tract (right) | 70 | 15 | A0B1 | N |
| 8 | 73 | F | Control\* | Lymphomatosis cerebri | NA | NA | NA | Ottawa | Olfactory bulb (both) | ~24 | 49 | NA | N |
| | | | | | | | | | Olfactory tract (both) | | | | |
| 9 | 75 | F | Control | No significant neuropathology | NA | Lung cancer and CAD | NA | Ottawa | Olfactory bulb + tract | ~72 | 8 | NA | N |
| 10 | 77 | M | Control\* | Meningioma | NA | NA | NA | Ottawa | Olfactory tract | ~72 | 8 | NA | N |
| 11 | 78 | M | Control | Metastasized esophageal cancer; mediastinitis/pleuritis; cardiac hypertrophy | O2 nasal | Heart failure due to advanced cancer and mediastinitis | 9 | St. Gallen | Olfactory bulb + tract (right) | 22 | 12 | A1B2 | N |
| 12 | 79 | F | Control | No significant neuropathology | NA | NA | NA | Ottawa | Olfactory bulb | ~72 | 53 | NA | N |
| | | | | | | | | | Olfactory tract (both) | | | | |
| 13 | 79 | F | Control | Severe aortic stenosis; cardiac hypertrophy | No | Heart failure | 10 | St. Gallen | Olfactory bulb + tract (right) | 36 | 13 | A1B1 | N |
| | | | | | | | | | Olfactory bulb + tract (left) | | | | |
| 14 | 79 | F | Control\* | Craniocerebral trauma | NA | NA | NA | Ottawa | Olfactory bulb | ~48 | 50 | NA | N |
| | | | | | | | | | Olfactory bulb + tract | | | | |
| 15 | 80 | M | Control | AML infiltration of spleen, liver and kidneys; epicarditis, pericarditis | No | Respiratory and kidney failure due to AML | 9 | St. Gallen | Olfactory bulb + tract | 39.5 | 4 | NA | N |
| 16 | 84 | F | Control\* | Acute hemorrhagic leukoencephalopathy | NA | NA | NA | Ottawa | Olfactory bulb + tract (both) | 24 | 29 | NA | N |
| 17 | 51 | M | COVID19+ | Bacterial pneumonia; DAD | ECMO | Respiratory failure | 27 | St. Gallen | Olfactory bulb + tract (right) | 23 | 14 | A0B1 | N |
| | | | | | | | | | Olfactory bulb + tract (left) | | | | |
| 18 | 52 | M | COVID19+ | Cerebrovascular disease + Covid | NA | NA | NA | Ottawa | Olfactory bulb | ~72 | 94 | NA | N |
| | | | | | | | | | Olfactory tract | | | | |
| 19 | 56 | M | COVID19+ | Pulmonary embolism; chronic myocarditis, cardiac hypertrophy; organizing pneumonia; old stroke lesion | Intubation | Respiratory failure | 5 | St. Gallen | Olfactory bulb + tract (right) | 33 | 37 | A0B1 | N |
| | | | | | | | | | Olfactory bulb + tract (left) | | | | |
| 20 | 59 | M | COVID19+ | Severe liver cirrhosis; viral pneumonia; cardiac hypertrophy | Intubation (ICU) and O2 nasal (ward) | Unknown | 49 | St. Gallen | Olfactory bulb + tract (right) | 39 | 16 | NA | N |
| | | | | | | | | | Olfactory bulb + tract (left) | | | | |
| 21 | 61 | F | COVID19+ | Pleural hemorrhage, minimal organizing pneumonia; AITL with heart involvement | O2 nasal | Respiratory failure | 7 | St. Gallen | Olfactory bulb + tract (right) | 15 | 15 | NA | N |
| | | | | | | | | | Olfactory bulb + tract (left) | | | | |
| 22 | 63 | F | COVID19+ | Pulmonary embolism; bacterial pneumonia; organizing DAD | NIV | Pulmonary embolism | 14 | St. Gallen | Olfactory bulb + tract (right) | 24 | 14 | A0B1 | N |
| | | | | | | | | | Olfactory tract (left) | | | | |
| 23 | 73 | F | COVID19+ | Retroperitoneal hemorrhage, pulmonary embolism with lung infarction; cardiac hypertrophy | O2 nasal | Hemorrhagic shock | 5 | St. Gallen | Olfactory bulb + tract (right) | 64 | 48 | A2B1 | N |
| | | | | | | | | | Olfactory bulb + tract (left) | | | | |
| 24 | 76 | M | COVID19+ | DAD; organizing pneumonia; leg vein thrombosis | No | Respiratory failure | 11 | St. Gallen | Olfactory bulb + tract (right) | 29 | 8 | NA | N |
| | | | | | | | | | Olfactory bulb + tract (left) | | | | |
| 25 | 76 | F | COVID19+ | Retroperitoneal hemorrhage; DAD; bacterial pneumonia | O2 nasal | Hemorrhagic shock | 7 | St. Gallen | Olfactory bulb + tract (right) | 23 | 12 | A0B2 | N |
| | | | | | | | | | Olfactory bulb + tract (left) | | | | |
Supplemental Table 1: Detailed characteristics of all subjects examined at autopsy.
Characteristics listed include age (in years); sex (F, female; M, male); diagnostic group (HCO, healthy control; NCO, neurological control; LBD, Lewy body disease; AD, Alzheimer disease; OND, other neurological disease); pathology diagnosis at autopsy; ventilation; clinical cause of death; number of days in hospital; site of tissue collection; tissue analyzed; post mortem interval (PMI; in hours); hippocampal (hippocamp.) staining; a-synuclein staining in the Substantia nigra (SN) and dorsal motor nucleus of the vagus nerve (DNV). * Indicate cases with an inflammatory condition. # Indicate cases with mixed pathology.

## Slide 4
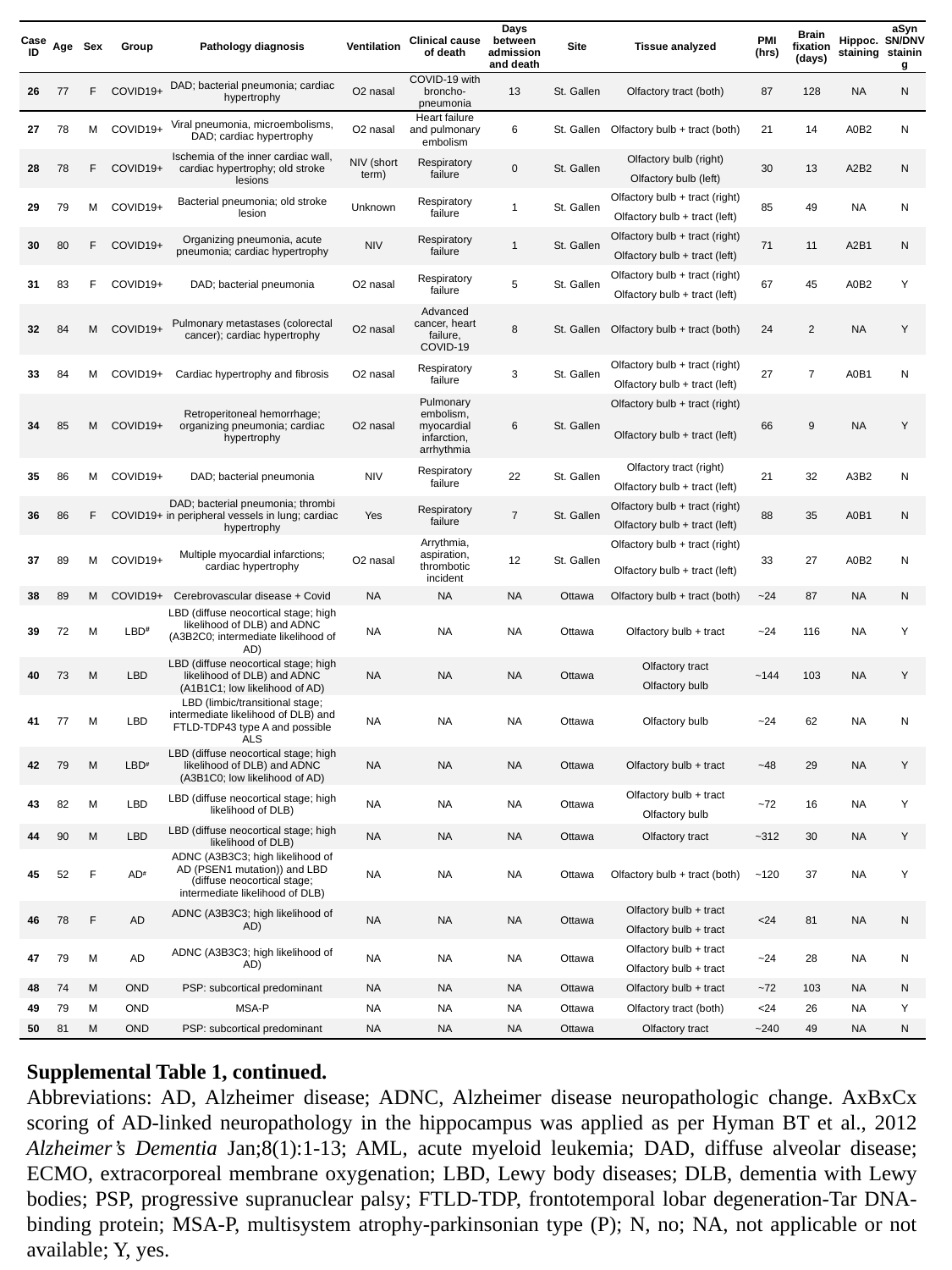

| Case ID | Age | Sex | Group | Pathology diagnosis | Ventilation | Clinical cause of death | Days between admission and death | Site | Tissue analyzed | PMI (hrs) | Brain fixation (days) | Hippoc. staining | aSyn SN/DNV staining |
| --- | --- | --- | --- | --- | --- | --- | --- | --- | --- | --- | --- | --- | --- |
| 26 | 77 | F | COVID19+ | DAD; bacterial pneumonia; cardiac hypertrophy | O2 nasal | COVID-19 with broncho-pneumonia | 13 | St. Gallen | Olfactory tract (both) | 87 | 128 | NA | N |
| 27 | 78 | M | COVID19+ | Viral pneumonia, microembolisms, DAD; cardiac hypertrophy | O2 nasal | Heart failure and pulmonary embolism | 6 | St. Gallen | Olfactory bulb + tract (both) | 21 | 14 | A0B2 | N |
| 28 | 78 | F | COVID19+ | Ischemia of the inner cardiac wall, cardiac hypertrophy; old stroke lesions | NIV (short term) | Respiratory failure | 0 | St. Gallen | Olfactory bulb (right) | 30 | 13 | A2B2 | N |
| | | | | | | | | | Olfactory bulb (left) | | | | |
| 29 | 79 | M | COVID19+ | Bacterial pneumonia; old stroke lesion | Unknown | Respiratory failure | 1 | St. Gallen | Olfactory bulb + tract (right) | 85 | 49 | NA | N |
| | | | | | | | | | Olfactory bulb + tract (left) | | | | |
| 30 | 80 | F | COVID19+ | Organizing pneumonia, acute pneumonia; cardiac hypertrophy | NIV | Respiratory failure | 1 | St. Gallen | Olfactory bulb + tract (right) | 71 | 11 | A2B1 | N |
| | | | | | | | | | Olfactory bulb + tract (left) | | | | |
| 31 | 83 | F | COVID19+ | DAD; bacterial pneumonia | O2 nasal | Respiratory failure | 5 | St. Gallen | Olfactory bulb + tract (right) | 67 | 45 | A0B2 | Y |
| | | | | | | | | | Olfactory bulb + tract (left) | | | | |
| 32 | 84 | M | COVID19+ | Pulmonary metastases (colorectal cancer); cardiac hypertrophy | O2 nasal | Advanced cancer, heart failure, COVID-19 | 8 | St. Gallen | Olfactory bulb + tract (both) | 24 | 2 | NA | Y |
| 33 | 84 | M | COVID19+ | Cardiac hypertrophy and fibrosis | O2 nasal | Respiratory failure | 3 | St. Gallen | Olfactory bulb + tract (right) | 27 | 7 | A0B1 | N |
| | | | | | | | | | Olfactory bulb + tract (left) | | | | |
| 34 | 85 | M | COVID19+ | Retroperitoneal hemorrhage; organizing pneumonia; cardiac hypertrophy | O2 nasal | Pulmonary embolism, myocardial infarction, arrhythmia | 6 | St. Gallen | Olfactory bulb + tract (right) | 66 | 9 | NA | Y |
| | | | | | | | | | Olfactory bulb + tract (left) | | | | |
| 35 | 86 | M | COVID19+ | DAD; bacterial pneumonia | NIV | Respiratory failure | 22 | St. Gallen | Olfactory tract (right) | 21 | 32 | A3B2 | N |
| | | | | | | | | | Olfactory bulb + tract (left) | | | | |
| 36 | 86 | F | COVID19+ | DAD; bacterial pneumonia; thrombi in peripheral vessels in lung; cardiac hypertrophy | Yes | Respiratory failure | 7 | St. Gallen | Olfactory bulb + tract (right) | 88 | 35 | A0B1 | N |
| | | | | | | | | | Olfactory bulb + tract (left) | | | | |
| 37 | 89 | M | COVID19+ | Multiple myocardial infarctions; cardiac hypertrophy | O2 nasal | Arrythmia, aspiration, thrombotic incident | 12 | St. Gallen | Olfactory bulb + tract (right) | 33 | 27 | A0B2 | N |
| | | | | | | | | | Olfactory bulb + tract (left) | | | | |
| 38 | 89 | M | COVID19+ | Cerebrovascular disease + Covid | NA | NA | NA | Ottawa | Olfactory bulb + tract (both) | ~24 | 87 | NA | N |
| 39 | 72 | M | LBD# | LBD (diffuse neocortical stage; high likelihood of DLB) and ADNC (A3B2C0; intermediate likelihood of AD) | NA | NA | NA | Ottawa | Olfactory bulb + tract | ~24 | 116 | NA | Y |
| 40 | 73 | M | LBD | LBD (diffuse neocortical stage; high likelihood of DLB) and ADNC (A1B1C1; low likelihood of AD) | NA | NA | NA | Ottawa | Olfactory tract | ~144 | 103 | NA | Y |
| | | | | | | | | | Olfactory bulb | | | | |
| 41 | 77 | M | LBD | LBD (limbic/transitional stage; intermediate likelihood of DLB) and FTLD-TDP43 type A and possible ALS | NA | NA | NA | Ottawa | Olfactory bulb | ~24 | 62 | NA | N |
| 42 | 79 | M | LBD# | LBD (diffuse neocortical stage; high likelihood of DLB) and ADNC (A3B1C0; low likelihood of AD) | NA | NA | NA | Ottawa | Olfactory bulb + tract | ~48 | 29 | NA | Y |
| 43 | 82 | M | LBD | LBD (diffuse neocortical stage; high likelihood of DLB) | NA | NA | NA | Ottawa | Olfactory bulb + tract | ~72 | 16 | NA | Y |
| | | | | | | | | | Olfactory bulb | | | | |
| 44 | 90 | M | LBD | LBD (diffuse neocortical stage; high likelihood of DLB) | NA | NA | NA | Ottawa | Olfactory tract | ~312 | 30 | NA | Y |
| 45 | 52 | F | AD# | ADNC (A3B3C3; high likelihood of AD (PSEN1 mutation)) and LBD (diffuse neocortical stage; intermediate likelihood of DLB) | NA | NA | NA | Ottawa | Olfactory bulb + tract (both) | ~120 | 37 | NA | Y |
| 46 | 78 | F | AD | ADNC (A3B3C3; high likelihood of AD) | NA | NA | NA | Ottawa | Olfactory bulb + tract | <24 | 81 | NA | N |
| | | | | | | | | | Olfactory bulb + tract | | | | |
| 47 | 79 | M | AD | ADNC (A3B3C3; high likelihood of AD) | NA | NA | NA | Ottawa | Olfactory bulb + tract | ~24 | 28 | NA | N |
| | | | | | | | | | Olfactory bulb + tract | | | | |
| 48 | 74 | M | OND | PSP: subcortical predominant | NA | NA | NA | Ottawa | Olfactory bulb + tract | ~72 | 103 | NA | N |
| 49 | 79 | M | OND | MSA-P | NA | NA | NA | Ottawa | Olfactory tract (both) | <24 | 26 | NA | Y |
| 50 | 81 | M | OND | PSP: subcortical predominant | NA | NA | NA | Ottawa | Olfactory tract | ~240 | 49 | NA | N |
Supplemental Table 1, continued.
Abbreviations: AD, Alzheimer disease; ADNC, Alzheimer disease neuropathologic change. AxBxCx scoring of AD-linked neuropathology in the hippocampus was applied as per Hyman BT et al., 2012 Alzheimer’s Dementia Jan;8(1):1-13; AML, acute myeloid leukemia; DAD, diffuse alveolar disease; ECMO, extracorporeal membrane oxygenation; LBD, Lewy body diseases; DLB, dementia with Lewy bodies; PSP, progressive supranuclear palsy; FTLD-TDP, frontotemporal lobar degeneration-Tar DNA-binding protein; MSA-P, multisystem atrophy-parkinsonian type (P); N, no; NA, not applicable or not available; Y, yes.

## Slide 5
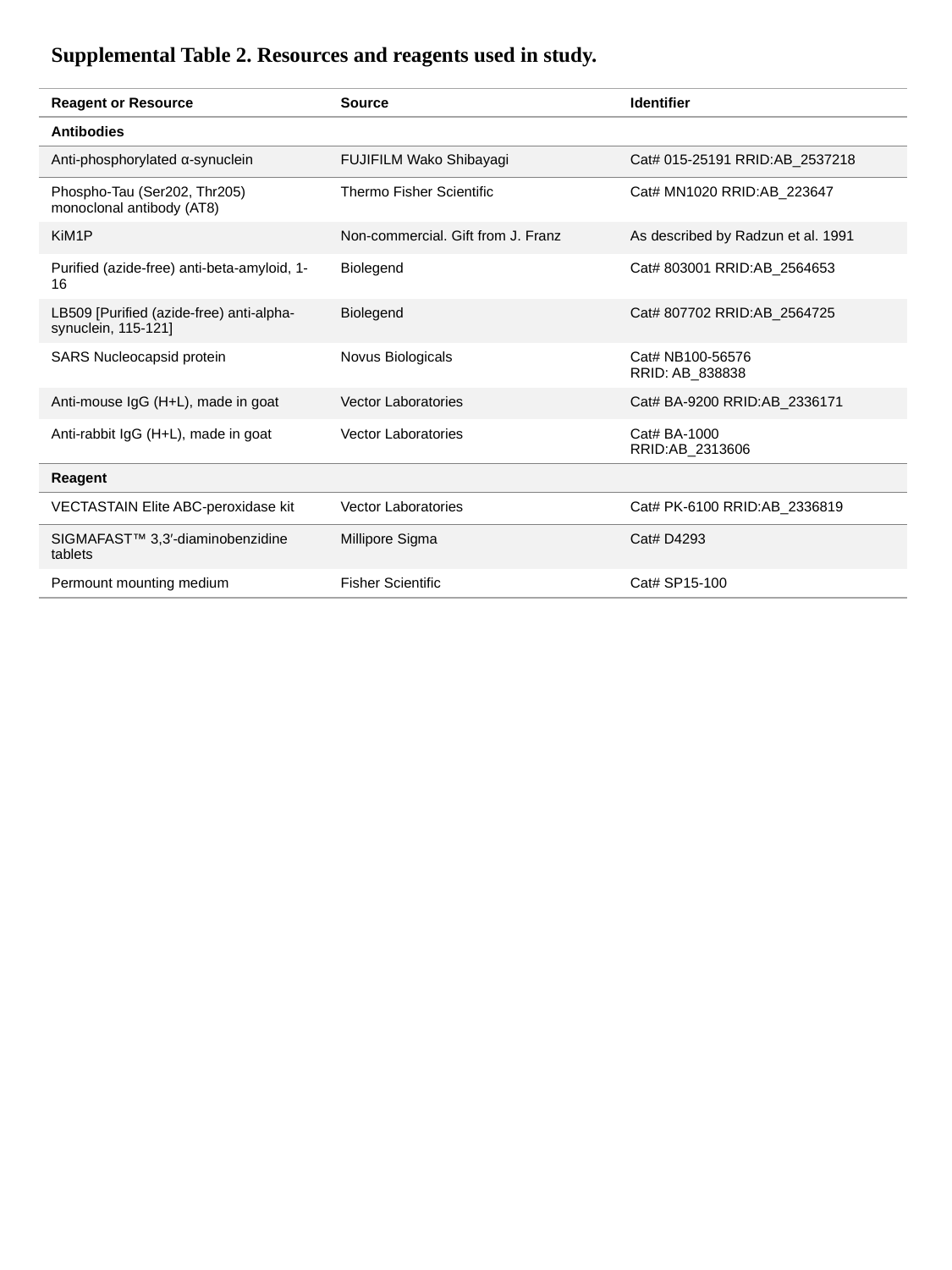

Supplemental Table 2. Resources and reagents used in study.
| Reagent or Resource | Source | Identifier |
| --- | --- | --- |
| Antibodies | | |
| Anti-phosphorylated α-synuclein | FUJIFILM Wako Shibayagi | Cat# 015-25191 RRID:AB\_2537218 |
| Phospho-Tau (Ser202, Thr205) monoclonal antibody (AT8) | Thermo Fisher Scientific | Cat# MN1020 RRID:AB\_223647 |
| KiM1P | Non-commercial. Gift from J. Franz | As described by Radzun et al. 1991 |
| Purified (azide-free) anti-beta-amyloid, 1-16 | Biolegend | Cat# 803001 RRID:AB\_2564653 |
| LB509 [Purified (azide-free) anti-alpha-synuclein, 115-121] | Biolegend | Cat# 807702 RRID:AB\_2564725 |
| SARS Nucleocapsid protein | Novus Biologicals | Cat# NB100-56576 RRID: AB\_838838 |
| Anti-mouse IgG (H+L), made in goat | Vector Laboratories | Cat# BA-9200 RRID:AB\_2336171 |
| Anti-rabbit IgG (H+L), made in goat | Vector Laboratories | Cat# BA-1000 RRID:AB\_2313606 |
| Reagent | | |
| VECTASTAIN Elite ABC-peroxidase kit | Vector Laboratories | Cat# PK-6100 RRID:AB\_2336819 |
| SIGMAFAST™ 3,3′-diaminobenzidine tablets | Millipore Sigma | Cat# D4293 |
| Permount mounting medium | Fisher Scientific | Cat# SP15-100 |

## Slide 6
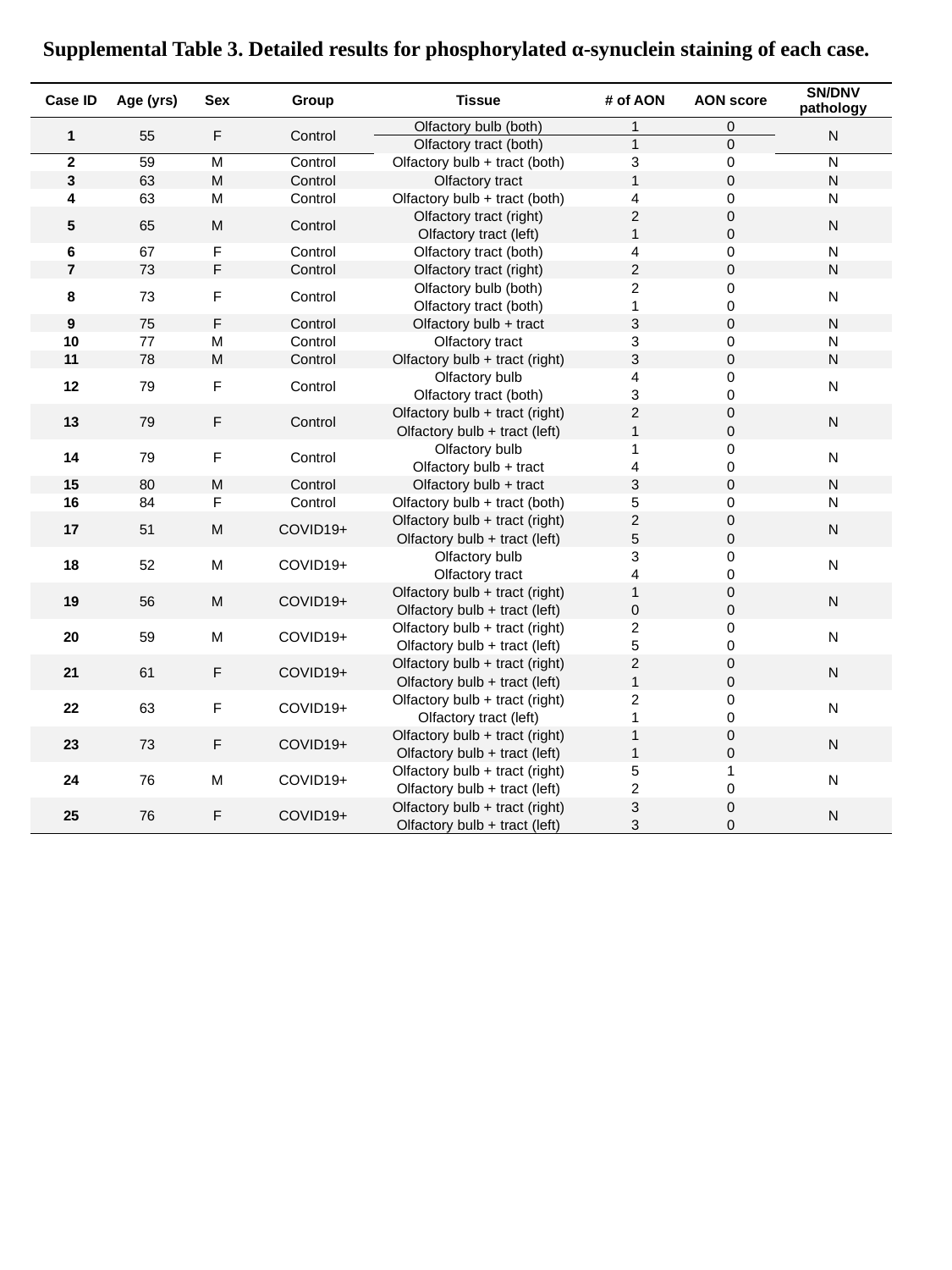

Supplemental Table 3. Detailed results for phosphorylated α-synuclein staining of each case.
| Case ID | Age (yrs) | Sex | Group | Tissue | # of AON | AON score | SN/DNV pathology |
| --- | --- | --- | --- | --- | --- | --- | --- |
| 1 | 55 | F | Control | Olfactory bulb (both) | 1 | 0 | N |
| | | | | Olfactory tract (both) | 1 | 0 | |
| 2 | 59 | M | Control | Olfactory bulb + tract (both) | 3 | 0 | N |
| 3 | 63 | M | Control | Olfactory tract | 1 | 0 | N |
| 4 | 63 | M | Control | Olfactory bulb + tract (both) | 4 | 0 | N |
| 5 | 65 | M | Control | Olfactory tract (right) | 2 | 0 | N |
| | | | | Olfactory tract (left) | 1 | 0 | |
| 6 | 67 | F | Control | Olfactory tract (both) | 4 | 0 | N |
| 7 | 73 | F | Control | Olfactory tract (right) | 2 | 0 | N |
| 8 | 73 | F | Control | Olfactory bulb (both) | 2 | 0 | N |
| | | | | Olfactory tract (both) | 1 | 0 | |
| 9 | 75 | F | Control | Olfactory bulb + tract | 3 | 0 | N |
| 10 | 77 | M | Control | Olfactory tract | 3 | 0 | N |
| 11 | 78 | M | Control | Olfactory bulb + tract (right) | 3 | 0 | N |
| 12 | 79 | F | Control | Olfactory bulb | 4 | 0 | N |
| | | | | Olfactory tract (both) | 3 | 0 | |
| 13 | 79 | F | Control | Olfactory bulb + tract (right) | 2 | 0 | N |
| | | | | Olfactory bulb + tract (left) | 1 | 0 | |
| 14 | 79 | F | Control | Olfactory bulb | 1 | 0 | N |
| | | | | Olfactory bulb + tract | 4 | 0 | |
| 15 | 80 | M | Control | Olfactory bulb + tract | 3 | 0 | N |
| 16 | 84 | F | Control | Olfactory bulb + tract (both) | 5 | 0 | N |
| 17 | 51 | M | COVID19+ | Olfactory bulb + tract (right) | 2 | 0 | N |
| | | | | Olfactory bulb + tract (left) | 5 | 0 | |
| 18 | 52 | M | COVID19+ | Olfactory bulb | 3 | 0 | N |
| | | | | Olfactory tract | 4 | 0 | |
| 19 | 56 | M | COVID19+ | Olfactory bulb + tract (right) | 1 | 0 | N |
| | | | | Olfactory bulb + tract (left) | 0 | 0 | |
| 20 | 59 | M | COVID19+ | Olfactory bulb + tract (right) | 2 | 0 | N |
| | | | | Olfactory bulb + tract (left) | 5 | 0 | |
| 21 | 61 | F | COVID19+ | Olfactory bulb + tract (right) | 2 | 0 | N |
| | | | | Olfactory bulb + tract (left) | 1 | 0 | |
| 22 | 63 | F | COVID19+ | Olfactory bulb + tract (right) | 2 | 0 | N |
| | | | | Olfactory tract (left) | 1 | 0 | |
| 23 | 73 | F | COVID19+ | Olfactory bulb + tract (right) | 1 | 0 | N |
| | | | | Olfactory bulb + tract (left) | 1 | 0 | |
| 24 | 76 | M | COVID19+ | Olfactory bulb + tract (right) | 5 | 1 | N |
| | | | | Olfactory bulb + tract (left) | 2 | 0 | |
| 25 | 76 | F | COVID19+ | Olfactory bulb + tract (right) | 3 | 0 | N |
| | | | | Olfactory bulb + tract (left) | 3 | 0 | |

## Slide 7
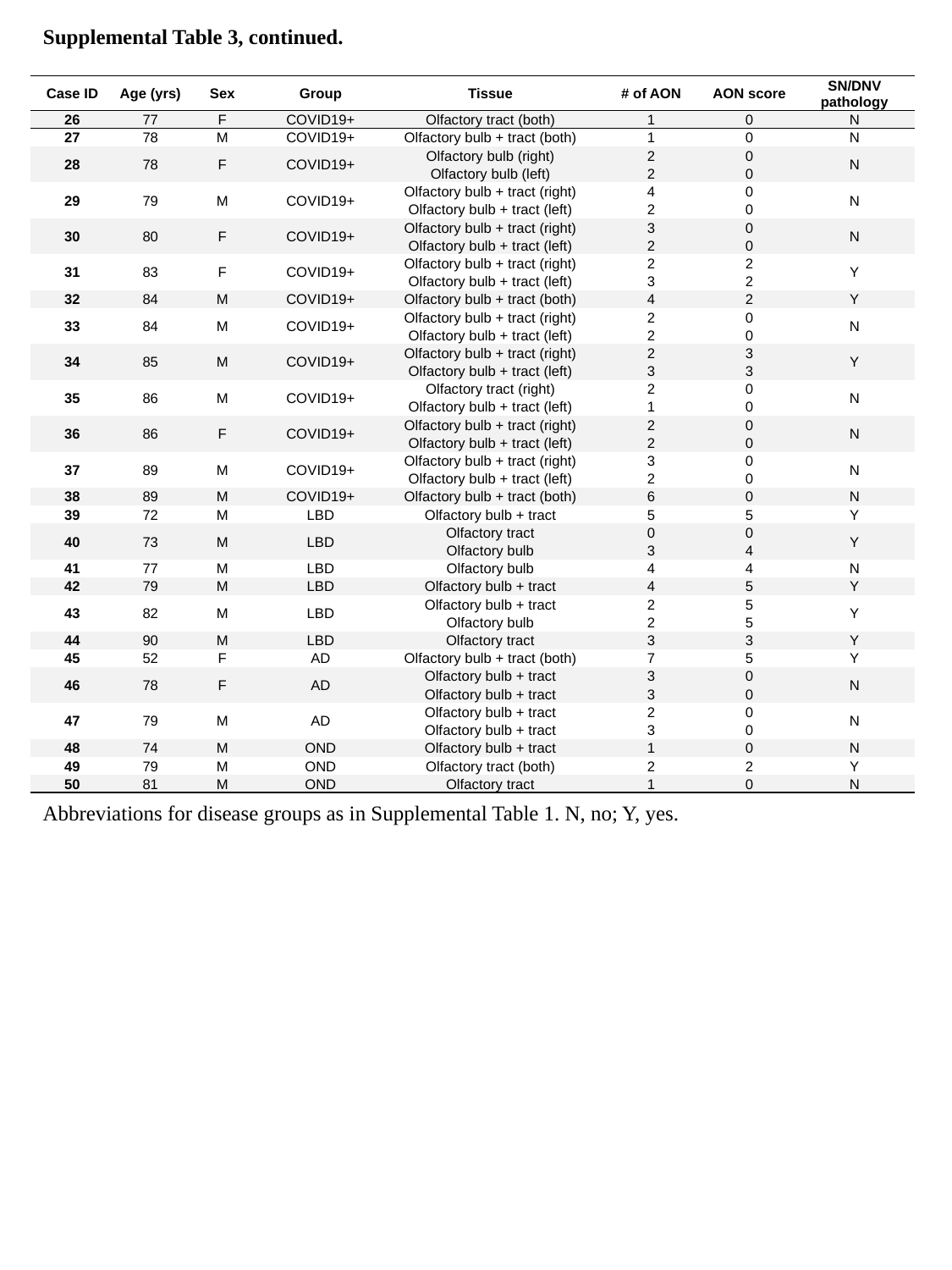

Supplemental Table 3, continued.
| Case ID | Age (yrs) | Sex | Group | Tissue | # of AON | AON score | SN/DNV pathology |
| --- | --- | --- | --- | --- | --- | --- | --- |
| 26 | 77 | F | COVID19+ | Olfactory tract (both) | 1 | 0 | N |
| 27 | 78 | M | COVID19+ | Olfactory bulb + tract (both) | 1 | 0 | N |
| 28 | 78 | F | COVID19+ | Olfactory bulb (right) | 2 | 0 | N |
| | | | | Olfactory bulb (left) | 2 | 0 | |
| 29 | 79 | M | COVID19+ | Olfactory bulb + tract (right) | 4 | 0 | N |
| | | | | Olfactory bulb + tract (left) | 2 | 0 | |
| 30 | 80 | F | COVID19+ | Olfactory bulb + tract (right) | 3 | 0 | N |
| | | | | Olfactory bulb + tract (left) | 2 | 0 | |
| 31 | 83 | F | COVID19+ | Olfactory bulb + tract (right) | 2 | 2 | Y |
| | | | | Olfactory bulb + tract (left) | 3 | 2 | |
| 32 | 84 | M | COVID19+ | Olfactory bulb + tract (both) | 4 | 2 | Y |
| 33 | 84 | M | COVID19+ | Olfactory bulb + tract (right) | 2 | 0 | N |
| | | | | Olfactory bulb + tract (left) | 2 | 0 | |
| 34 | 85 | M | COVID19+ | Olfactory bulb + tract (right) | 2 | 3 | Y |
| | | | | Olfactory bulb + tract (left) | 3 | 3 | |
| 35 | 86 | M | COVID19+ | Olfactory tract (right) | 2 | 0 | N |
| | | | | Olfactory bulb + tract (left) | 1 | 0 | |
| 36 | 86 | F | COVID19+ | Olfactory bulb + tract (right) | 2 | 0 | N |
| | | | | Olfactory bulb + tract (left) | 2 | 0 | |
| 37 | 89 | M | COVID19+ | Olfactory bulb + tract (right) | 3 | 0 | N |
| | | | | Olfactory bulb + tract (left) | 2 | 0 | |
| 38 | 89 | M | COVID19+ | Olfactory bulb + tract (both) | 6 | 0 | N |
| 39 | 72 | M | LBD | Olfactory bulb + tract | 5 | 5 | Y |
| 40 | 73 | M | LBD | Olfactory tract | 0 | 0 | Y |
| | | | | Olfactory bulb | 3 | 4 | |
| 41 | 77 | M | LBD | Olfactory bulb | 4 | 4 | N |
| 42 | 79 | M | LBD | Olfactory bulb + tract | 4 | 5 | Y |
| 43 | 82 | M | LBD | Olfactory bulb + tract | 2 | 5 | Y |
| | | | | Olfactory bulb | 2 | 5 | |
| 44 | 90 | M | LBD | Olfactory tract | 3 | 3 | Y |
| 45 | 52 | F | AD | Olfactory bulb + tract (both) | 7 | 5 | Y |
| 46 | 78 | F | AD | Olfactory bulb + tract | 3 | 0 | N |
| | | | | Olfactory bulb + tract | 3 | 0 | |
| 47 | 79 | M | AD | Olfactory bulb + tract | 2 | 0 | N |
| | | | | Olfactory bulb + tract | 3 | 0 | |
| 48 | 74 | M | OND | Olfactory bulb + tract | 1 | 0 | N |
| 49 | 79 | M | OND | Olfactory tract (both) | 2 | 2 | Y |
| 50 | 81 | M | OND | Olfactory tract | 1 | 0 | N |
Abbreviations for disease groups as in Supplemental Table 1. N, no; Y, yes.

## Slide 8
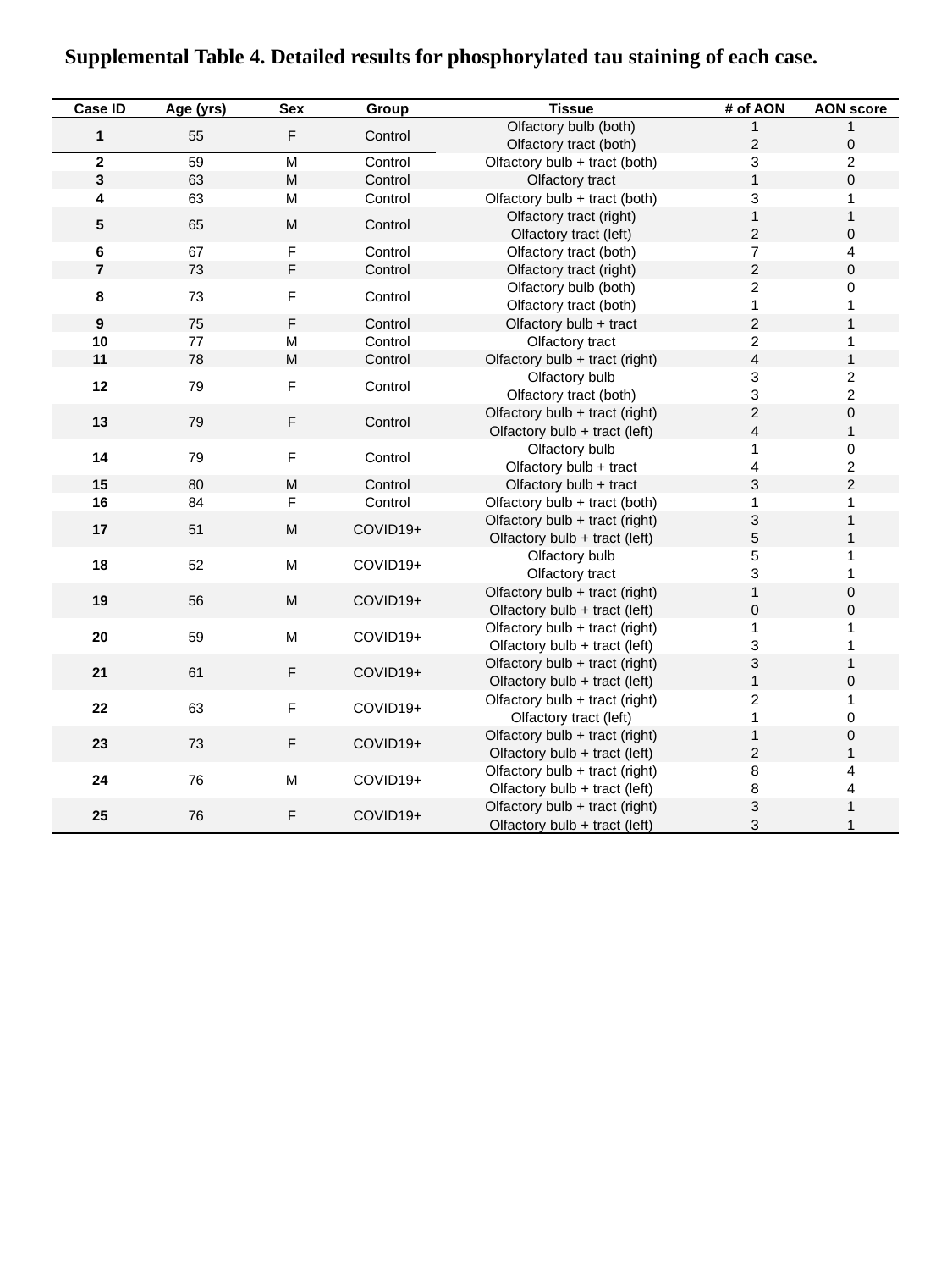

Supplemental Table 4. Detailed results for phosphorylated tau staining of each case.
| Case ID | Age (yrs) | Sex | Group | Tissue | # of AON | AON score |
| --- | --- | --- | --- | --- | --- | --- |
| 1 | 55 | F | Control | Olfactory bulb (both) | 1 | 1 |
| | | | | Olfactory tract (both) | 2 | 0 |
| 2 | 59 | M | Control | Olfactory bulb + tract (both) | 3 | 2 |
| 3 | 63 | M | Control | Olfactory tract | 1 | 0 |
| 4 | 63 | M | Control | Olfactory bulb + tract (both) | 3 | 1 |
| 5 | 65 | M | Control | Olfactory tract (right) | 1 | 1 |
| | | | | Olfactory tract (left) | 2 | 0 |
| 6 | 67 | F | Control | Olfactory tract (both) | 7 | 4 |
| 7 | 73 | F | Control | Olfactory tract (right) | 2 | 0 |
| 8 | 73 | F | Control | Olfactory bulb (both) | 2 | 0 |
| | | | | Olfactory tract (both) | 1 | 1 |
| 9 | 75 | F | Control | Olfactory bulb + tract | 2 | 1 |
| 10 | 77 | M | Control | Olfactory tract | 2 | 1 |
| 11 | 78 | M | Control | Olfactory bulb + tract (right) | 4 | 1 |
| 12 | 79 | F | Control | Olfactory bulb | 3 | 2 |
| | | | | Olfactory tract (both) | 3 | 2 |
| 13 | 79 | F | Control | Olfactory bulb + tract (right) | 2 | 0 |
| | | | | Olfactory bulb + tract (left) | 4 | 1 |
| 14 | 79 | F | Control | Olfactory bulb | 1 | 0 |
| | | | | Olfactory bulb + tract | 4 | 2 |
| 15 | 80 | M | Control | Olfactory bulb + tract | 3 | 2 |
| 16 | 84 | F | Control | Olfactory bulb + tract (both) | 1 | 1 |
| 17 | 51 | M | COVID19+ | Olfactory bulb + tract (right) | 3 | 1 |
| | | | | Olfactory bulb + tract (left) | 5 | 1 |
| 18 | 52 | M | COVID19+ | Olfactory bulb | 5 | 1 |
| | | | | Olfactory tract | 3 | 1 |
| 19 | 56 | M | COVID19+ | Olfactory bulb + tract (right) | 1 | 0 |
| | | | | Olfactory bulb + tract (left) | 0 | 0 |
| 20 | 59 | M | COVID19+ | Olfactory bulb + tract (right) | 1 | 1 |
| | | | | Olfactory bulb + tract (left) | 3 | 1 |
| 21 | 61 | F | COVID19+ | Olfactory bulb + tract (right) | 3 | 1 |
| | | | | Olfactory bulb + tract (left) | 1 | 0 |
| 22 | 63 | F | COVID19+ | Olfactory bulb + tract (right) | 2 | 1 |
| | | | | Olfactory tract (left) | 1 | 0 |
| 23 | 73 | F | COVID19+ | Olfactory bulb + tract (right) | 1 | 0 |
| | | | | Olfactory bulb + tract (left) | 2 | 1 |
| 24 | 76 | M | COVID19+ | Olfactory bulb + tract (right) | 8 | 4 |
| | | | | Olfactory bulb + tract (left) | 8 | 4 |
| 25 | 76 | F | COVID19+ | Olfactory bulb + tract (right) | 3 | 1 |
| | | | | Olfactory bulb + tract (left) | 3 | 1 |

## Slide 9
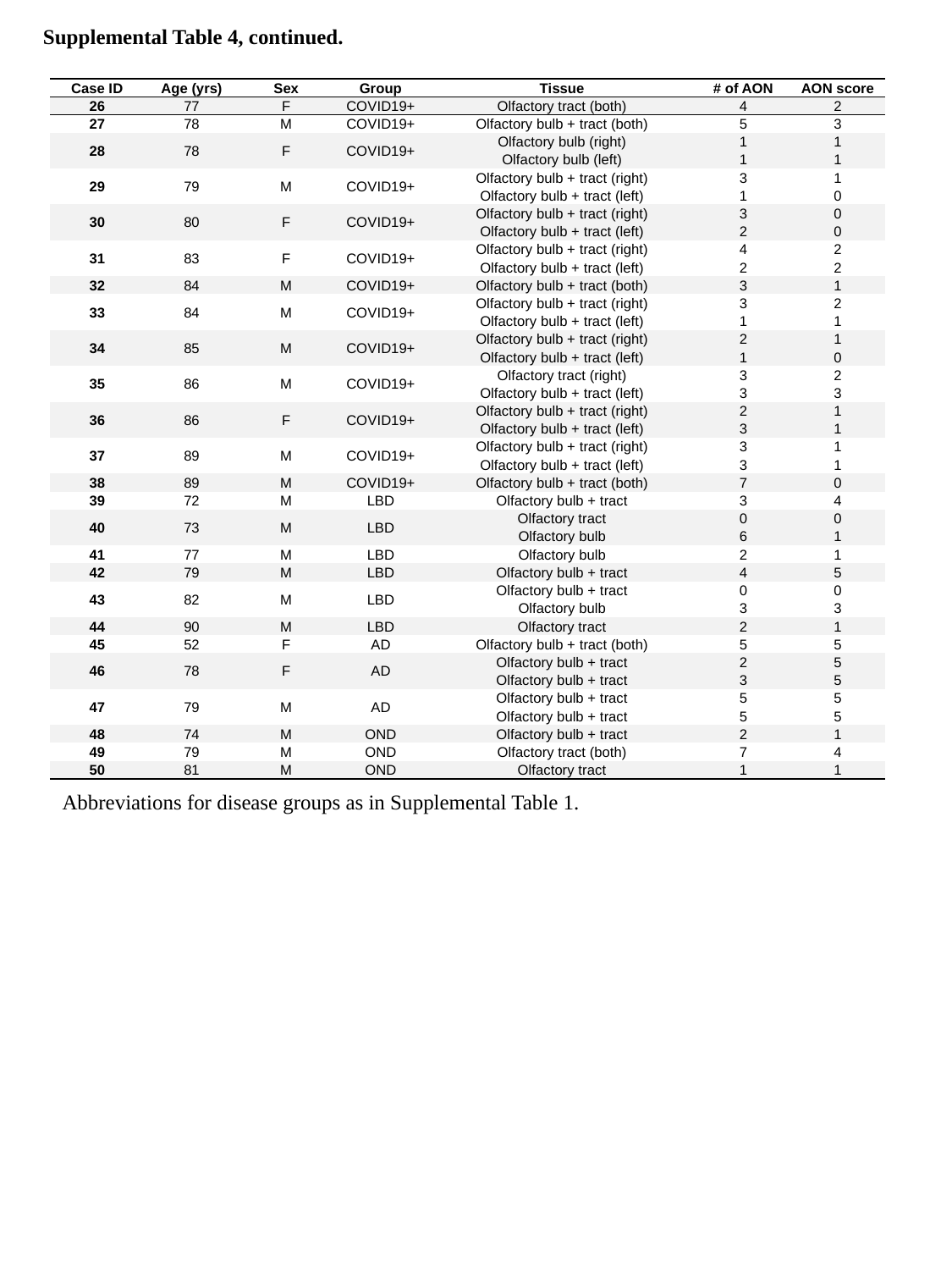

Supplemental Table 4, continued.
| Case ID | Age (yrs) | Sex | Group | Tissue | # of AON | AON score |
| --- | --- | --- | --- | --- | --- | --- |
| 26 | 77 | F | COVID19+ | Olfactory tract (both) | 4 | 2 |
| 27 | 78 | M | COVID19+ | Olfactory bulb + tract (both) | 5 | 3 |
| 28 | 78 | F | COVID19+ | Olfactory bulb (right) | 1 | 1 |
| | | | | Olfactory bulb (left) | 1 | 1 |
| 29 | 79 | M | COVID19+ | Olfactory bulb + tract (right) | 3 | 1 |
| | | | | Olfactory bulb + tract (left) | 1 | 0 |
| 30 | 80 | F | COVID19+ | Olfactory bulb + tract (right) | 3 | 0 |
| | | | | Olfactory bulb + tract (left) | 2 | 0 |
| 31 | 83 | F | COVID19+ | Olfactory bulb + tract (right) | 4 | 2 |
| | | | | Olfactory bulb + tract (left) | 2 | 2 |
| 32 | 84 | M | COVID19+ | Olfactory bulb + tract (both) | 3 | 1 |
| 33 | 84 | M | COVID19+ | Olfactory bulb + tract (right) | 3 | 2 |
| | | | | Olfactory bulb + tract (left) | 1 | 1 |
| 34 | 85 | M | COVID19+ | Olfactory bulb + tract (right) | 2 | 1 |
| | | | | Olfactory bulb + tract (left) | 1 | 0 |
| 35 | 86 | M | COVID19+ | Olfactory tract (right) | 3 | 2 |
| | | | | Olfactory bulb + tract (left) | 3 | 3 |
| 36 | 86 | F | COVID19+ | Olfactory bulb + tract (right) | 2 | 1 |
| | | | | Olfactory bulb + tract (left) | 3 | 1 |
| 37 | 89 | M | COVID19+ | Olfactory bulb + tract (right) | 3 | 1 |
| | | | | Olfactory bulb + tract (left) | 3 | 1 |
| 38 | 89 | M | COVID19+ | Olfactory bulb + tract (both) | 7 | 0 |
| 39 | 72 | M | LBD | Olfactory bulb + tract | 3 | 4 |
| 40 | 73 | M | LBD | Olfactory tract | 0 | 0 |
| | | | | Olfactory bulb | 6 | 1 |
| 41 | 77 | M | LBD | Olfactory bulb | 2 | 1 |
| 42 | 79 | M | LBD | Olfactory bulb + tract | 4 | 5 |
| 43 | 82 | M | LBD | Olfactory bulb + tract | 0 | 0 |
| | | | | Olfactory bulb | 3 | 3 |
| 44 | 90 | M | LBD | Olfactory tract | 2 | 1 |
| 45 | 52 | F | AD | Olfactory bulb + tract (both) | 5 | 5 |
| 46 | 78 | F | AD | Olfactory bulb + tract | 2 | 5 |
| | | | | Olfactory bulb + tract | 3 | 5 |
| 47 | 79 | M | AD | Olfactory bulb + tract | 5 | 5 |
| | | | | Olfactory bulb + tract | 5 | 5 |
| 48 | 74 | M | OND | Olfactory bulb + tract | 2 | 1 |
| 49 | 79 | M | OND | Olfactory tract (both) | 7 | 4 |
| 50 | 81 | M | OND | Olfactory tract | 1 | 1 |
Abbreviations for disease groups as in Supplemental Table 1.

## Slide 10
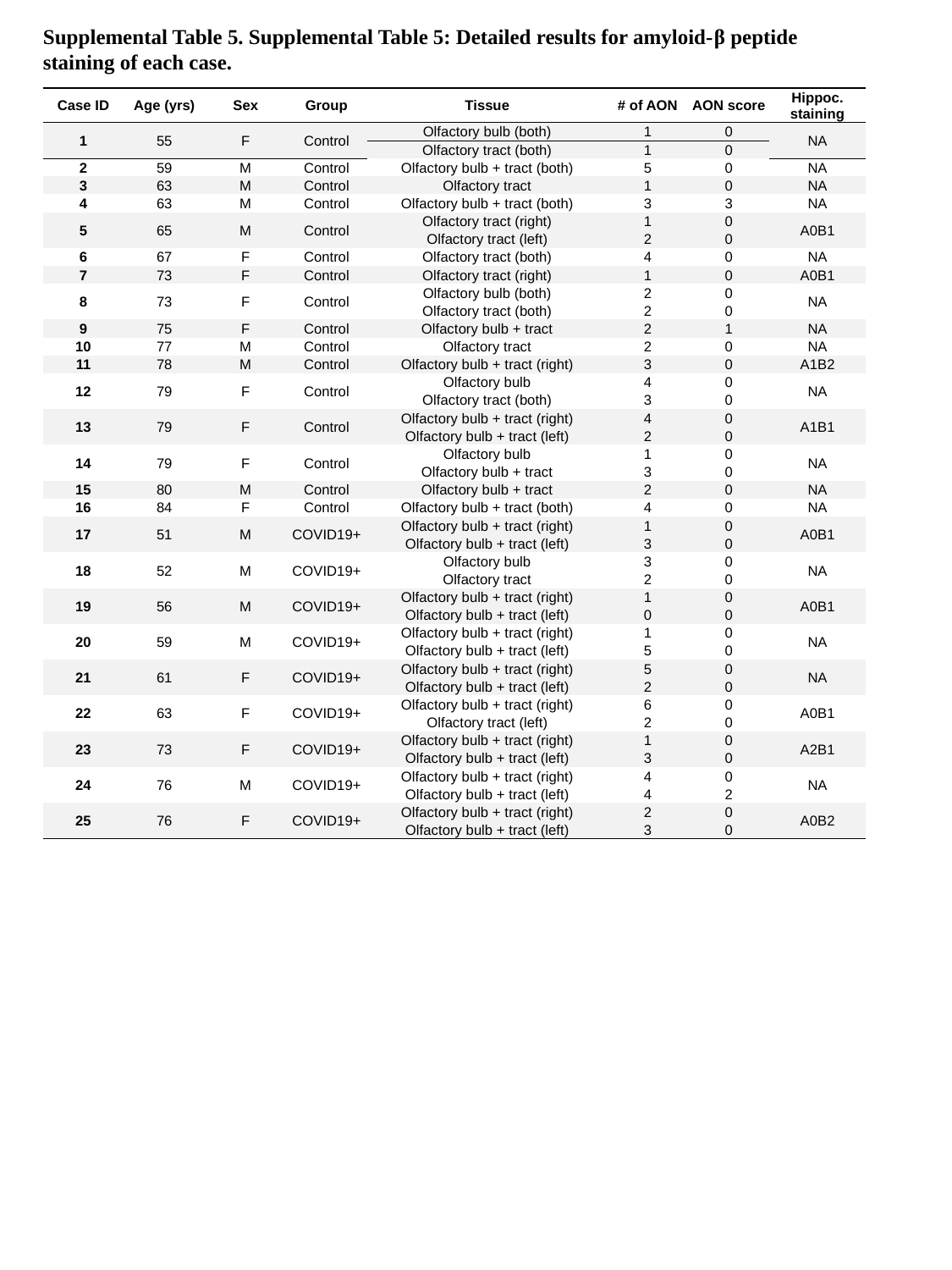

Supplemental Table 5. Supplemental Table 5: Detailed results for amyloid-β peptide staining of each case.
| Case ID | Age (yrs) | Sex | Group | Tissue | # of AON | AON score | Hippoc. staining |
| --- | --- | --- | --- | --- | --- | --- | --- |
| 1 | 55 | F | Control | Olfactory bulb (both) | 1 | 0 | NA |
| | | | | Olfactory tract (both) | 1 | 0 | |
| 2 | 59 | M | Control | Olfactory bulb + tract (both) | 5 | 0 | NA |
| 3 | 63 | M | Control | Olfactory tract | 1 | 0 | NA |
| 4 | 63 | M | Control | Olfactory bulb + tract (both) | 3 | 3 | NA |
| 5 | 65 | M | Control | Olfactory tract (right) | 1 | 0 | A0B1 |
| | | | | Olfactory tract (left) | 2 | 0 | |
| 6 | 67 | F | Control | Olfactory tract (both) | 4 | 0 | NA |
| 7 | 73 | F | Control | Olfactory tract (right) | 1 | 0 | A0B1 |
| 8 | 73 | F | Control | Olfactory bulb (both) | 2 | 0 | NA |
| | | | | Olfactory tract (both) | 2 | 0 | |
| 9 | 75 | F | Control | Olfactory bulb + tract | 2 | 1 | NA |
| 10 | 77 | M | Control | Olfactory tract | 2 | 0 | NA |
| 11 | 78 | M | Control | Olfactory bulb + tract (right) | 3 | 0 | A1B2 |
| 12 | 79 | F | Control | Olfactory bulb | 4 | 0 | NA |
| | | | | Olfactory tract (both) | 3 | 0 | |
| 13 | 79 | F | Control | Olfactory bulb + tract (right) | 4 | 0 | A1B1 |
| | | | | Olfactory bulb + tract (left) | 2 | 0 | |
| 14 | 79 | F | Control | Olfactory bulb | 1 | 0 | NA |
| | | | | Olfactory bulb + tract | 3 | 0 | |
| 15 | 80 | M | Control | Olfactory bulb + tract | 2 | 0 | NA |
| 16 | 84 | F | Control | Olfactory bulb + tract (both) | 4 | 0 | NA |
| 17 | 51 | M | COVID19+ | Olfactory bulb + tract (right) | 1 | 0 | A0B1 |
| | | | | Olfactory bulb + tract (left) | 3 | 0 | |
| 18 | 52 | M | COVID19+ | Olfactory bulb | 3 | 0 | NA |
| | | | | Olfactory tract | 2 | 0 | |
| 19 | 56 | M | COVID19+ | Olfactory bulb + tract (right) | 1 | 0 | A0B1 |
| | | | | Olfactory bulb + tract (left) | 0 | 0 | |
| 20 | 59 | M | COVID19+ | Olfactory bulb + tract (right) | 1 | 0 | NA |
| | | | | Olfactory bulb + tract (left) | 5 | 0 | |
| 21 | 61 | F | COVID19+ | Olfactory bulb + tract (right) | 5 | 0 | NA |
| | | | | Olfactory bulb + tract (left) | 2 | 0 | |
| 22 | 63 | F | COVID19+ | Olfactory bulb + tract (right) | 6 | 0 | A0B1 |
| | | | | Olfactory tract (left) | 2 | 0 | |
| 23 | 73 | F | COVID19+ | Olfactory bulb + tract (right) | 1 | 0 | A2B1 |
| | | | | Olfactory bulb + tract (left) | 3 | 0 | |
| 24 | 76 | M | COVID19+ | Olfactory bulb + tract (right) | 4 | 0 | NA |
| | | | | Olfactory bulb + tract (left) | 4 | 2 | |
| 25 | 76 | F | COVID19+ | Olfactory bulb + tract (right) | 2 | 0 | A0B2 |
| | | | | Olfactory bulb + tract (left) | 3 | 0 | |

## Slide 11
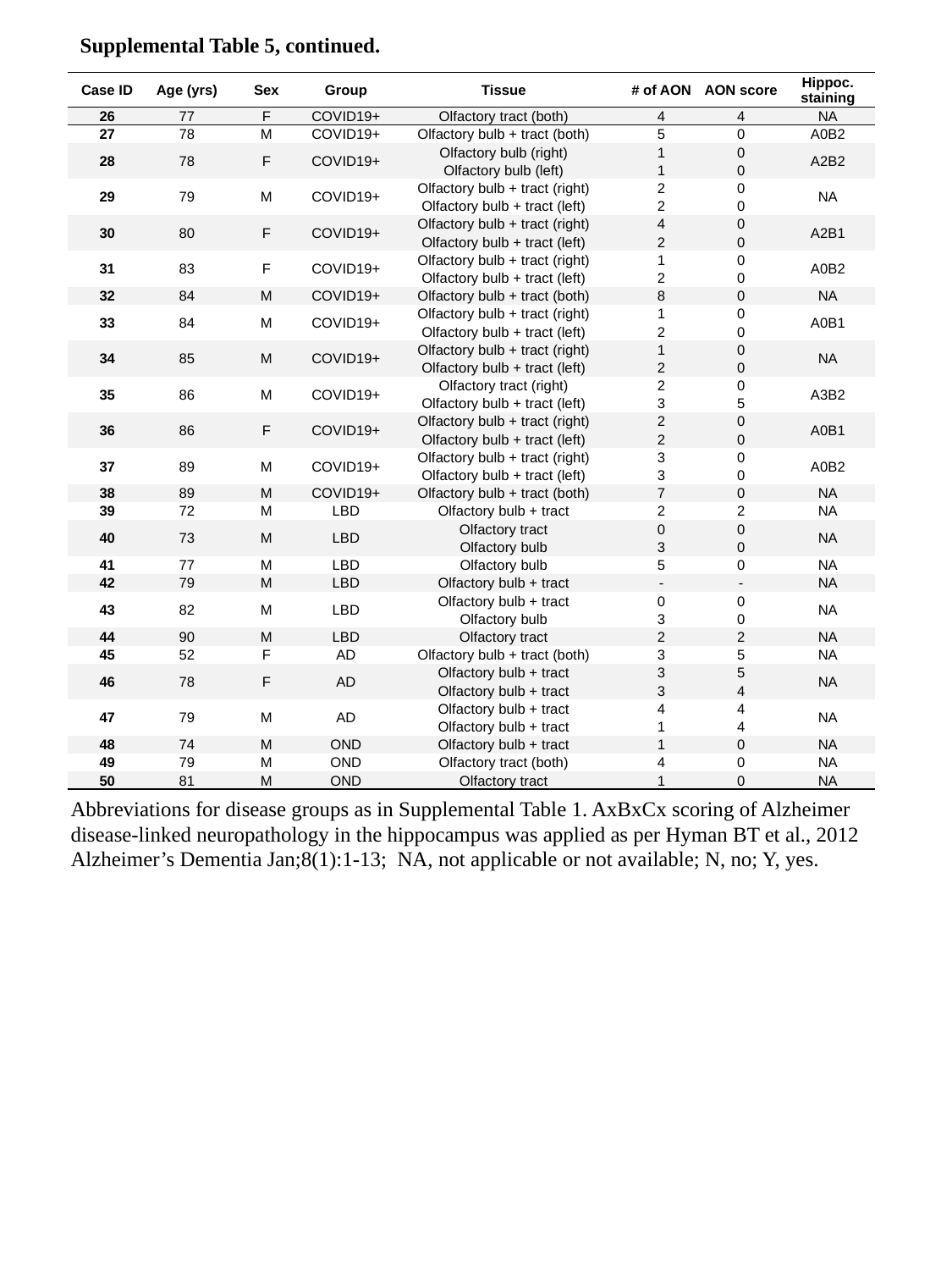

Supplemental Table 5, continued.
| Case ID | Age (yrs) | Sex | Group | Tissue | # of AON | AON score | Hippoc. staining |
| --- | --- | --- | --- | --- | --- | --- | --- |
| 26 | 77 | F | COVID19+ | Olfactory tract (both) | 4 | 4 | NA |
| 27 | 78 | M | COVID19+ | Olfactory bulb + tract (both) | 5 | 0 | A0B2 |
| 28 | 78 | F | COVID19+ | Olfactory bulb (right) | 1 | 0 | A2B2 |
| | | | | Olfactory bulb (left) | 1 | 0 | |
| 29 | 79 | M | COVID19+ | Olfactory bulb + tract (right) | 2 | 0 | NA |
| | | | | Olfactory bulb + tract (left) | 2 | 0 | |
| 30 | 80 | F | COVID19+ | Olfactory bulb + tract (right) | 4 | 0 | A2B1 |
| | | | | Olfactory bulb + tract (left) | 2 | 0 | |
| 31 | 83 | F | COVID19+ | Olfactory bulb + tract (right) | 1 | 0 | A0B2 |
| | | | | Olfactory bulb + tract (left) | 2 | 0 | |
| 32 | 84 | M | COVID19+ | Olfactory bulb + tract (both) | 8 | 0 | NA |
| 33 | 84 | M | COVID19+ | Olfactory bulb + tract (right) | 1 | 0 | A0B1 |
| | | | | Olfactory bulb + tract (left) | 2 | 0 | |
| 34 | 85 | M | COVID19+ | Olfactory bulb + tract (right) | 1 | 0 | NA |
| | | | | Olfactory bulb + tract (left) | 2 | 0 | |
| 35 | 86 | M | COVID19+ | Olfactory tract (right) | 2 | 0 | A3B2 |
| | | | | Olfactory bulb + tract (left) | 3 | 5 | |
| 36 | 86 | F | COVID19+ | Olfactory bulb + tract (right) | 2 | 0 | A0B1 |
| | | | | Olfactory bulb + tract (left) | 2 | 0 | |
| 37 | 89 | M | COVID19+ | Olfactory bulb + tract (right) | 3 | 0 | A0B2 |
| | | | | Olfactory bulb + tract (left) | 3 | 0 | |
| 38 | 89 | M | COVID19+ | Olfactory bulb + tract (both) | 7 | 0 | NA |
| 39 | 72 | M | LBD | Olfactory bulb + tract | 2 | 2 | NA |
| 40 | 73 | M | LBD | Olfactory tract | 0 | 0 | NA |
| | | | | Olfactory bulb | 3 | 0 | |
| 41 | 77 | M | LBD | Olfactory bulb | 5 | 0 | NA |
| 42 | 79 | M | LBD | Olfactory bulb + tract | - | - | NA |
| 43 | 82 | M | LBD | Olfactory bulb + tract | 0 | 0 | NA |
| | | | | Olfactory bulb | 3 | 0 | |
| 44 | 90 | M | LBD | Olfactory tract | 2 | 2 | NA |
| 45 | 52 | F | AD | Olfactory bulb + tract (both) | 3 | 5 | NA |
| 46 | 78 | F | AD | Olfactory bulb + tract | 3 | 5 | NA |
| | | | | Olfactory bulb + tract | 3 | 4 | |
| 47 | 79 | M | AD | Olfactory bulb + tract | 4 | 4 | NA |
| | | | | Olfactory bulb + tract | 1 | 4 | |
| 48 | 74 | M | OND | Olfactory bulb + tract | 1 | 0 | NA |
| 49 | 79 | M | OND | Olfactory tract (both) | 4 | 0 | NA |
| 50 | 81 | M | OND | Olfactory tract | 1 | 0 | NA |
Abbreviations for disease groups as in Supplemental Table 1. AxBxCx scoring of Alzheimer disease-linked neuropathology in the hippocampus was applied as per Hyman BT et al., 2012 Alzheimer’s Dementia Jan;8(1):1-13; NA, not applicable or not available; N, no; Y, yes.

## Slide 12
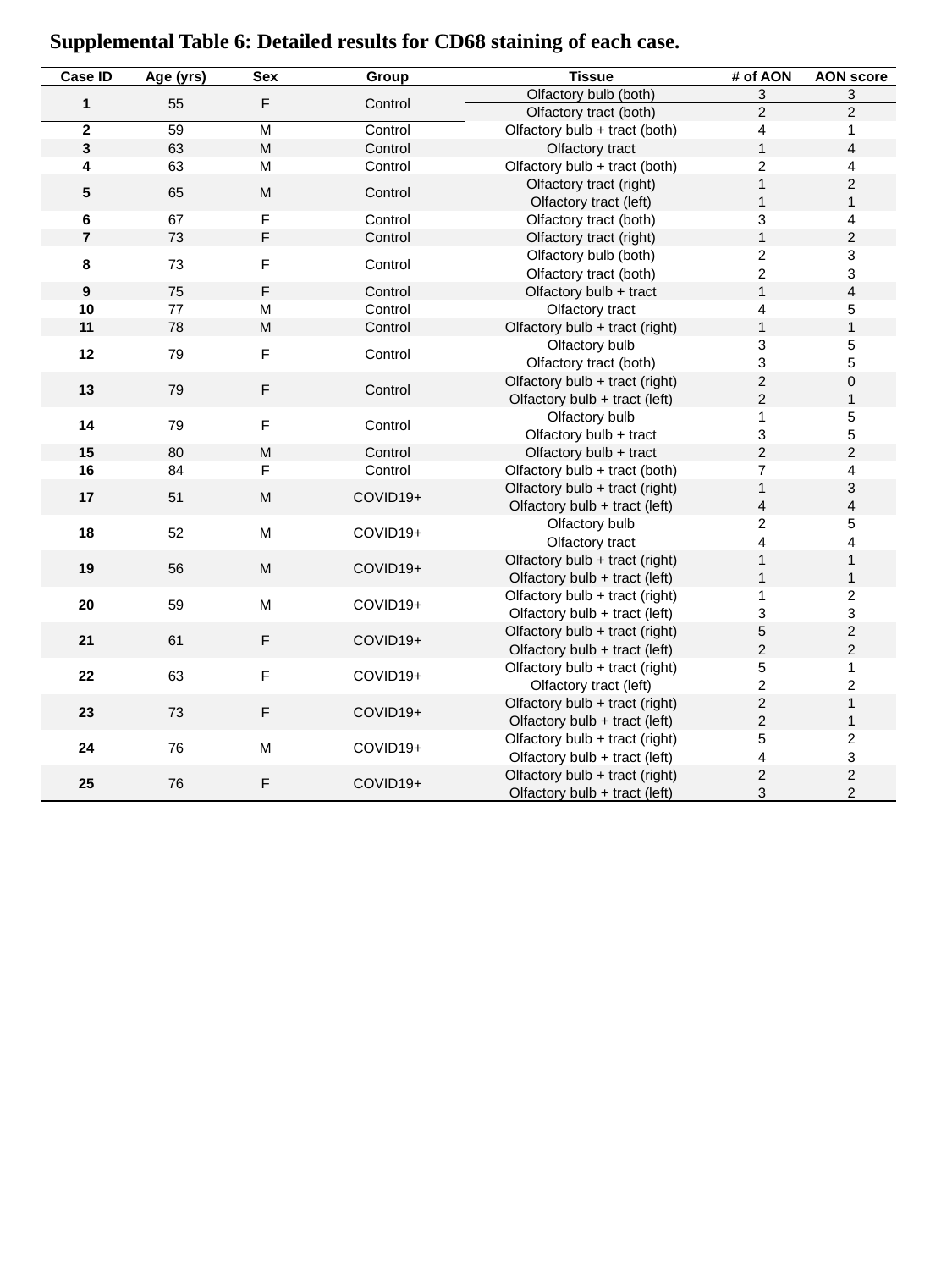

Supplemental Table 6: Detailed results for CD68 staining of each case.
| Case ID | Age (yrs) | Sex | Group | Tissue | # of AON | AON score |
| --- | --- | --- | --- | --- | --- | --- |
| 1 | 55 | F | Control | Olfactory bulb (both) | 3 | 3 |
| | | | | Olfactory tract (both) | 2 | 2 |
| 2 | 59 | M | Control | Olfactory bulb + tract (both) | 4 | 1 |
| 3 | 63 | M | Control | Olfactory tract | 1 | 4 |
| 4 | 63 | M | Control | Olfactory bulb + tract (both) | 2 | 4 |
| 5 | 65 | M | Control | Olfactory tract (right) | 1 | 2 |
| | | | | Olfactory tract (left) | 1 | 1 |
| 6 | 67 | F | Control | Olfactory tract (both) | 3 | 4 |
| 7 | 73 | F | Control | Olfactory tract (right) | 1 | 2 |
| 8 | 73 | F | Control | Olfactory bulb (both) | 2 | 3 |
| | | | | Olfactory tract (both) | 2 | 3 |
| 9 | 75 | F | Control | Olfactory bulb + tract | 1 | 4 |
| 10 | 77 | M | Control | Olfactory tract | 4 | 5 |
| 11 | 78 | M | Control | Olfactory bulb + tract (right) | 1 | 1 |
| 12 | 79 | F | Control | Olfactory bulb | 3 | 5 |
| | | | | Olfactory tract (both) | 3 | 5 |
| 13 | 79 | F | Control | Olfactory bulb + tract (right) | 2 | 0 |
| | | | | Olfactory bulb + tract (left) | 2 | 1 |
| 14 | 79 | F | Control | Olfactory bulb | 1 | 5 |
| | | | | Olfactory bulb + tract | 3 | 5 |
| 15 | 80 | M | Control | Olfactory bulb + tract | 2 | 2 |
| 16 | 84 | F | Control | Olfactory bulb + tract (both) | 7 | 4 |
| 17 | 51 | M | COVID19+ | Olfactory bulb + tract (right) | 1 | 3 |
| | | | | Olfactory bulb + tract (left) | 4 | 4 |
| 18 | 52 | M | COVID19+ | Olfactory bulb | 2 | 5 |
| | | | | Olfactory tract | 4 | 4 |
| 19 | 56 | M | COVID19+ | Olfactory bulb + tract (right) | 1 | 1 |
| | | | | Olfactory bulb + tract (left) | 1 | 1 |
| 20 | 59 | M | COVID19+ | Olfactory bulb + tract (right) | 1 | 2 |
| | | | | Olfactory bulb + tract (left) | 3 | 3 |
| 21 | 61 | F | COVID19+ | Olfactory bulb + tract (right) | 5 | 2 |
| | | | | Olfactory bulb + tract (left) | 2 | 2 |
| 22 | 63 | F | COVID19+ | Olfactory bulb + tract (right) | 5 | 1 |
| | | | | Olfactory tract (left) | 2 | 2 |
| 23 | 73 | F | COVID19+ | Olfactory bulb + tract (right) | 2 | 1 |
| | | | | Olfactory bulb + tract (left) | 2 | 1 |
| 24 | 76 | M | COVID19+ | Olfactory bulb + tract (right) | 5 | 2 |
| | | | | Olfactory bulb + tract (left) | 4 | 3 |
| 25 | 76 | F | COVID19+ | Olfactory bulb + tract (right) | 2 | 2 |
| | | | | Olfactory bulb + tract (left) | 3 | 2 |

## Slide 13
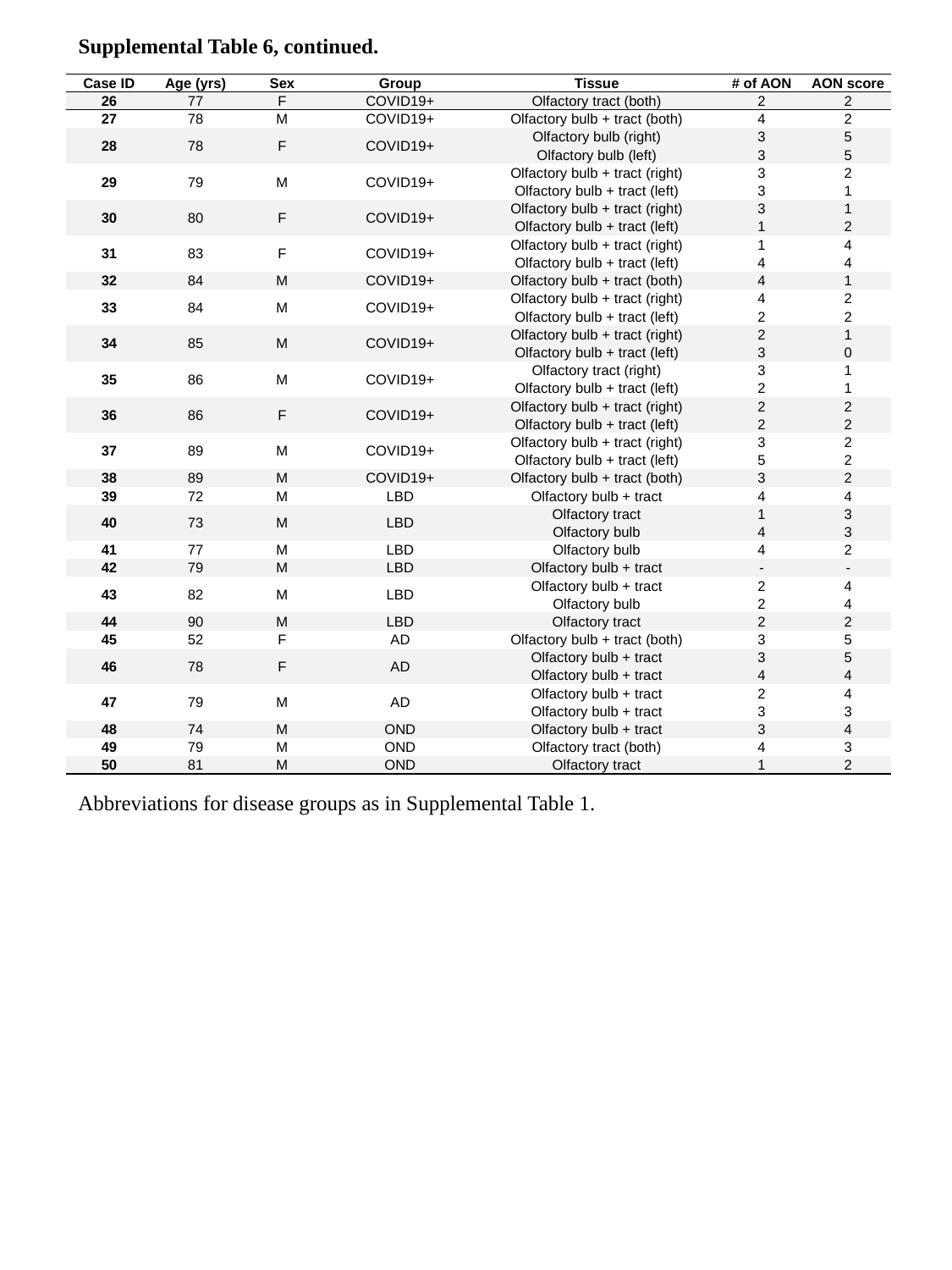

Supplemental Table 6, continued.
| Case ID | Age (yrs) | Sex | Group | Tissue | # of AON | AON score |
| --- | --- | --- | --- | --- | --- | --- |
| 26 | 77 | F | COVID19+ | Olfactory tract (both) | 2 | 2 |
| 27 | 78 | M | COVID19+ | Olfactory bulb + tract (both) | 4 | 2 |
| 28 | 78 | F | COVID19+ | Olfactory bulb (right) | 3 | 5 |
| | | | | Olfactory bulb (left) | 3 | 5 |
| 29 | 79 | M | COVID19+ | Olfactory bulb + tract (right) | 3 | 2 |
| | | | | Olfactory bulb + tract (left) | 3 | 1 |
| 30 | 80 | F | COVID19+ | Olfactory bulb + tract (right) | 3 | 1 |
| | | | | Olfactory bulb + tract (left) | 1 | 2 |
| 31 | 83 | F | COVID19+ | Olfactory bulb + tract (right) | 1 | 4 |
| | | | | Olfactory bulb + tract (left) | 4 | 4 |
| 32 | 84 | M | COVID19+ | Olfactory bulb + tract (both) | 4 | 1 |
| 33 | 84 | M | COVID19+ | Olfactory bulb + tract (right) | 4 | 2 |
| | | | | Olfactory bulb + tract (left) | 2 | 2 |
| 34 | 85 | M | COVID19+ | Olfactory bulb + tract (right) | 2 | 1 |
| | | | | Olfactory bulb + tract (left) | 3 | 0 |
| 35 | 86 | M | COVID19+ | Olfactory tract (right) | 3 | 1 |
| | | | | Olfactory bulb + tract (left) | 2 | 1 |
| 36 | 86 | F | COVID19+ | Olfactory bulb + tract (right) | 2 | 2 |
| | | | | Olfactory bulb + tract (left) | 2 | 2 |
| 37 | 89 | M | COVID19+ | Olfactory bulb + tract (right) | 3 | 2 |
| | | | | Olfactory bulb + tract (left) | 5 | 2 |
| 38 | 89 | M | COVID19+ | Olfactory bulb + tract (both) | 3 | 2 |
| 39 | 72 | M | LBD | Olfactory bulb + tract | 4 | 4 |
| 40 | 73 | M | LBD | Olfactory tract | 1 | 3 |
| | | | | Olfactory bulb | 4 | 3 |
| 41 | 77 | M | LBD | Olfactory bulb | 4 | 2 |
| 42 | 79 | M | LBD | Olfactory bulb + tract | - | - |
| 43 | 82 | M | LBD | Olfactory bulb + tract | 2 | 4 |
| | | | | Olfactory bulb | 2 | 4 |
| 44 | 90 | M | LBD | Olfactory tract | 2 | 2 |
| 45 | 52 | F | AD | Olfactory bulb + tract (both) | 3 | 5 |
| 46 | 78 | F | AD | Olfactory bulb + tract | 3 | 5 |
| | | | | Olfactory bulb + tract | 4 | 4 |
| 47 | 79 | M | AD | Olfactory bulb + tract | 2 | 4 |
| | | | | Olfactory bulb + tract | 3 | 3 |
| 48 | 74 | M | OND | Olfactory bulb + tract | 3 | 4 |
| 49 | 79 | M | OND | Olfactory tract (both) | 4 | 3 |
| 50 | 81 | M | OND | Olfactory tract | 1 | 2 |
Abbreviations for disease groups as in Supplemental Table 1.

## Slide 14
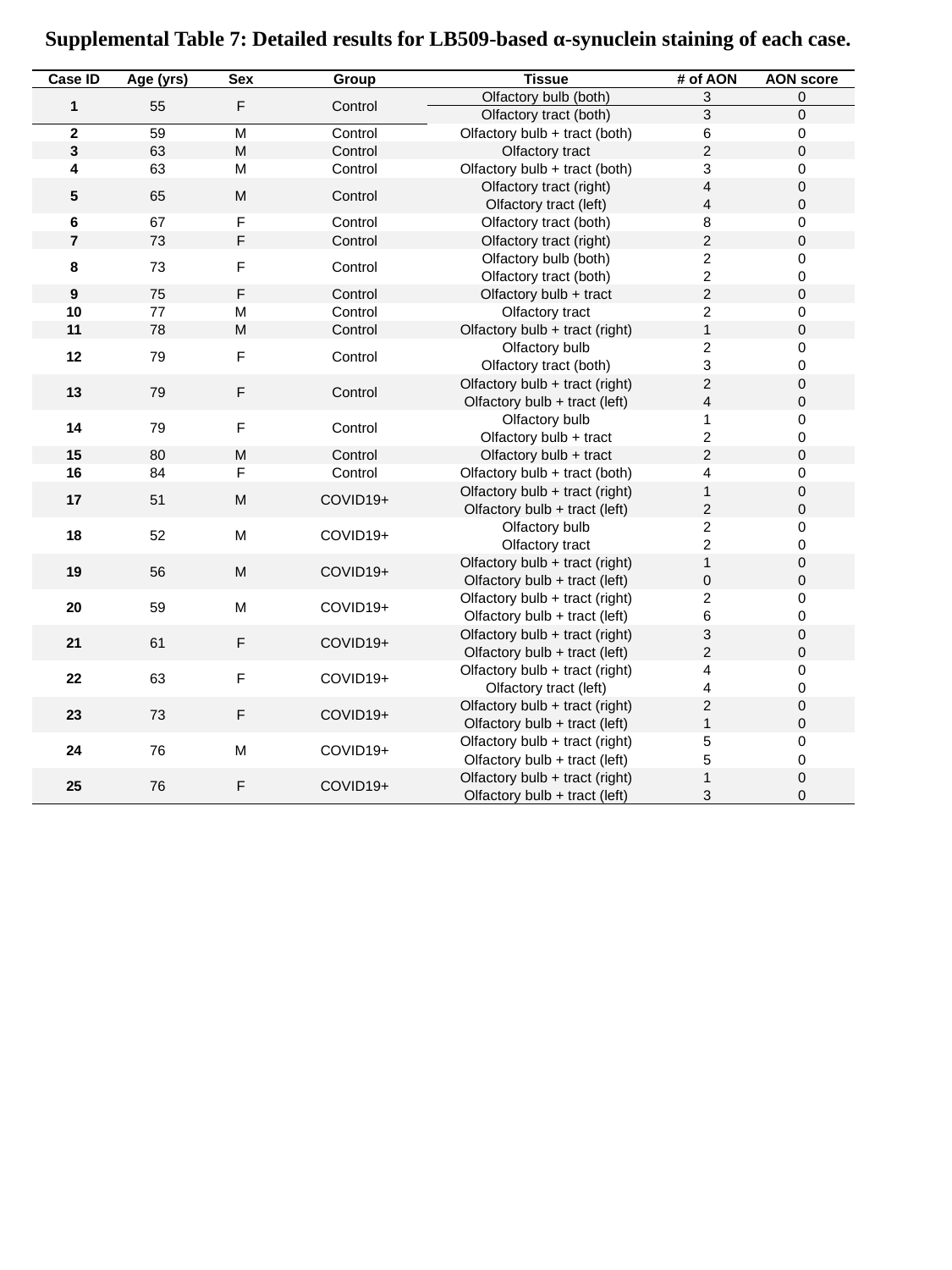

Supplemental Table 7: Detailed results for LB509-based α-synuclein staining of each case.
| Case ID | Age (yrs) | Sex | Group | Tissue | # of AON | AON score |
| --- | --- | --- | --- | --- | --- | --- |
| 1 | 55 | F | Control | Olfactory bulb (both) | 3 | 0 |
| | | | | Olfactory tract (both) | 3 | 0 |
| 2 | 59 | M | Control | Olfactory bulb + tract (both) | 6 | 0 |
| 3 | 63 | M | Control | Olfactory tract | 2 | 0 |
| 4 | 63 | M | Control | Olfactory bulb + tract (both) | 3 | 0 |
| 5 | 65 | M | Control | Olfactory tract (right) | 4 | 0 |
| | | | | Olfactory tract (left) | 4 | 0 |
| 6 | 67 | F | Control | Olfactory tract (both) | 8 | 0 |
| 7 | 73 | F | Control | Olfactory tract (right) | 2 | 0 |
| 8 | 73 | F | Control | Olfactory bulb (both) | 2 | 0 |
| | | | | Olfactory tract (both) | 2 | 0 |
| 9 | 75 | F | Control | Olfactory bulb + tract | 2 | 0 |
| 10 | 77 | M | Control | Olfactory tract | 2 | 0 |
| 11 | 78 | M | Control | Olfactory bulb + tract (right) | 1 | 0 |
| 12 | 79 | F | Control | Olfactory bulb | 2 | 0 |
| | | | | Olfactory tract (both) | 3 | 0 |
| 13 | 79 | F | Control | Olfactory bulb + tract (right) | 2 | 0 |
| | | | | Olfactory bulb + tract (left) | 4 | 0 |
| 14 | 79 | F | Control | Olfactory bulb | 1 | 0 |
| | | | | Olfactory bulb + tract | 2 | 0 |
| 15 | 80 | M | Control | Olfactory bulb + tract | 2 | 0 |
| 16 | 84 | F | Control | Olfactory bulb + tract (both) | 4 | 0 |
| 17 | 51 | M | COVID19+ | Olfactory bulb + tract (right) | 1 | 0 |
| | | | | Olfactory bulb + tract (left) | 2 | 0 |
| 18 | 52 | M | COVID19+ | Olfactory bulb | 2 | 0 |
| | | | | Olfactory tract | 2 | 0 |
| 19 | 56 | M | COVID19+ | Olfactory bulb + tract (right) | 1 | 0 |
| | | | | Olfactory bulb + tract (left) | 0 | 0 |
| 20 | 59 | M | COVID19+ | Olfactory bulb + tract (right) | 2 | 0 |
| | | | | Olfactory bulb + tract (left) | 6 | 0 |
| 21 | 61 | F | COVID19+ | Olfactory bulb + tract (right) | 3 | 0 |
| | | | | Olfactory bulb + tract (left) | 2 | 0 |
| 22 | 63 | F | COVID19+ | Olfactory bulb + tract (right) | 4 | 0 |
| | | | | Olfactory tract (left) | 4 | 0 |
| 23 | 73 | F | COVID19+ | Olfactory bulb + tract (right) | 2 | 0 |
| | | | | Olfactory bulb + tract (left) | 1 | 0 |
| 24 | 76 | M | COVID19+ | Olfactory bulb + tract (right) | 5 | 0 |
| | | | | Olfactory bulb + tract (left) | 5 | 0 |
| 25 | 76 | F | COVID19+ | Olfactory bulb + tract (right) | 1 | 0 |
| | | | | Olfactory bulb + tract (left) | 3 | 0 |

## Slide 15
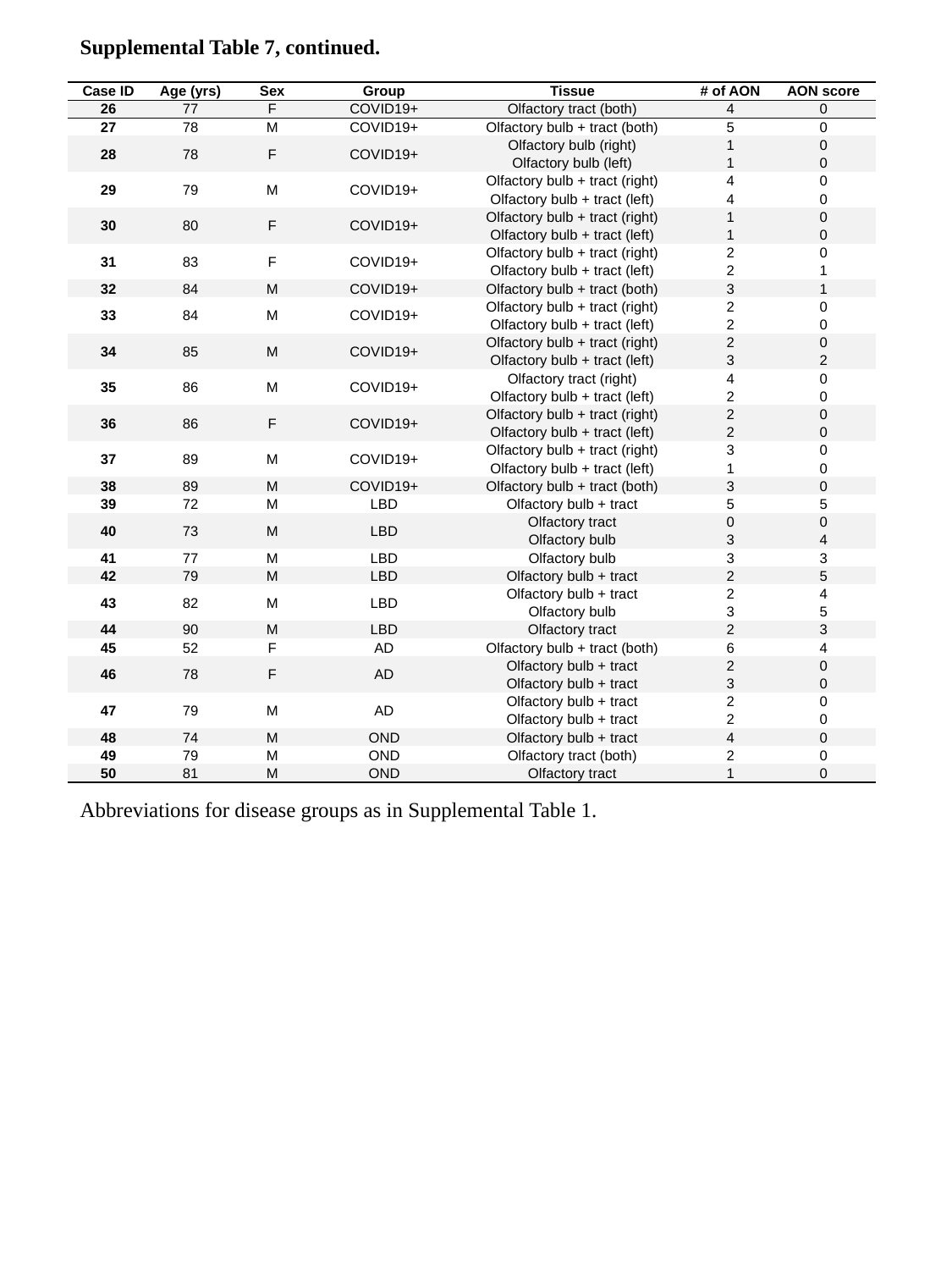

Supplemental Table 7, continued.
| Case ID | Age (yrs) | Sex | Group | Tissue | # of AON | AON score |
| --- | --- | --- | --- | --- | --- | --- |
| 26 | 77 | F | COVID19+ | Olfactory tract (both) | 4 | 0 |
| 27 | 78 | M | COVID19+ | Olfactory bulb + tract (both) | 5 | 0 |
| 28 | 78 | F | COVID19+ | Olfactory bulb (right) | 1 | 0 |
| | | | | Olfactory bulb (left) | 1 | 0 |
| 29 | 79 | M | COVID19+ | Olfactory bulb + tract (right) | 4 | 0 |
| | | | | Olfactory bulb + tract (left) | 4 | 0 |
| 30 | 80 | F | COVID19+ | Olfactory bulb + tract (right) | 1 | 0 |
| | | | | Olfactory bulb + tract (left) | 1 | 0 |
| 31 | 83 | F | COVID19+ | Olfactory bulb + tract (right) | 2 | 0 |
| | | | | Olfactory bulb + tract (left) | 2 | 1 |
| 32 | 84 | M | COVID19+ | Olfactory bulb + tract (both) | 3 | 1 |
| 33 | 84 | M | COVID19+ | Olfactory bulb + tract (right) | 2 | 0 |
| | | | | Olfactory bulb + tract (left) | 2 | 0 |
| 34 | 85 | M | COVID19+ | Olfactory bulb + tract (right) | 2 | 0 |
| | | | | Olfactory bulb + tract (left) | 3 | 2 |
| 35 | 86 | M | COVID19+ | Olfactory tract (right) | 4 | 0 |
| | | | | Olfactory bulb + tract (left) | 2 | 0 |
| 36 | 86 | F | COVID19+ | Olfactory bulb + tract (right) | 2 | 0 |
| | | | | Olfactory bulb + tract (left) | 2 | 0 |
| 37 | 89 | M | COVID19+ | Olfactory bulb + tract (right) | 3 | 0 |
| | | | | Olfactory bulb + tract (left) | 1 | 0 |
| 38 | 89 | M | COVID19+ | Olfactory bulb + tract (both) | 3 | 0 |
| 39 | 72 | M | LBD | Olfactory bulb + tract | 5 | 5 |
| 40 | 73 | M | LBD | Olfactory tract | 0 | 0 |
| | | | | Olfactory bulb | 3 | 4 |
| 41 | 77 | M | LBD | Olfactory bulb | 3 | 3 |
| 42 | 79 | M | LBD | Olfactory bulb + tract | 2 | 5 |
| 43 | 82 | M | LBD | Olfactory bulb + tract | 2 | 4 |
| | | | | Olfactory bulb | 3 | 5 |
| 44 | 90 | M | LBD | Olfactory tract | 2 | 3 |
| 45 | 52 | F | AD | Olfactory bulb + tract (both) | 6 | 4 |
| 46 | 78 | F | AD | Olfactory bulb + tract | 2 | 0 |
| | | | | Olfactory bulb + tract | 3 | 0 |
| 47 | 79 | M | AD | Olfactory bulb + tract | 2 | 0 |
| | | | | Olfactory bulb + tract | 2 | 0 |
| 48 | 74 | M | OND | Olfactory bulb + tract | 4 | 0 |
| 49 | 79 | M | OND | Olfactory tract (both) | 2 | 0 |
| 50 | 81 | M | OND | Olfactory tract | 1 | 0 |
Abbreviations for disease groups as in Supplemental Table 1.
